# Arrestin domain containing 3 promotes *Helicobacter pylori*–associated gastritis by regulating protease-activated receptor 1

**DOI:** 10.1172/jci.insight.135849

**Published:** 2020-08-06

**Authors:** Yu-gang Liu, Yong-sheng Teng, Zhi-guo Shan, Ping Cheng, Chuan-jie Hao, Yi-pin Lv, Fang-yuan Mao, Shi-ming Yang, Weisan Chen, Yong-liang Zhao, Nan You, Quan-ming Zou, Yuan Zhuang

**Affiliations:** 1 National Engineering Research Center of Immunological Products, Department of Microbiology and Biochemical Pharmacy, College of Pharmacy and Laboratory Medicine, Third Military Medical University, Chongqing, China.; 2 Department of Clinical Laboratory, the General Hospital of Western Theater Command, Chengdu, Sichuan, China.; 3 Department of General Surgery and Center of Minimal Invasive Gastrointestinal Surgery, Southwest Hospital, and; 4 Department of Gastroenterology, XinQiao Hospital, Third Military Medical University, Chongqing, China.; 5 La Trobe Institute of Molecular Science, La Trobe University, Bundoora, Victoria, Australia.; 6 Department of Hepatobiliary Surgery, XinQiao Hospital, Third Military Medical University, Chongqing, China.

**Keywords:** Gastroenterology, Infectious disease, Bacterial infections

## Abstract

Arrestin domain containing 3 (ARRDC3) represents a newly discovered α-arrestin involved in obesity, inflammation, and cancer. Here, we demonstrate a proinflammation role of ARRDC3 in *Helicobacter pylori*–associated gastritis. Increased ARRDC3 was detected in gastric mucosa of patients and mice infected with *H*. *pylori*. ARRDC3 in gastric epithelial cells (GECs) was induced by *H*. *pylori*, regulated by ERK and PI3K-AKT pathways in a *cagA*-dependent manner. Human gastric ARRDC3 correlated with the severity of gastritis, and mouse ARRDC3 from non-BM–derived cells promoted gastric inflammation. This inflammation was characterized by the CXCR2-dependent influx of CD45^+^CD11b^+^Ly6C^–^Ly6G^+^ neutrophils, whose migration was induced via the ARRDC3-dependent production of CXCL2 by GECs. Importantly, gastric inflammation was attenuated in *Arrdc3^–/–^* mice but increased in protease-activated receptor 1^–/–^ (*Par1*^–/–^) mice. Mechanistically, ARRDC3 in GECs directly interacted with PAR1 and negatively regulated PAR1 via ARRDC3-mediated lysosomal degradation, which abrogated the suppression of CXCL2 production and following neutrophil chemotaxis by PAR1, thereby contributing to the development of *H*. *pylori*–associated gastritis. This study identifies a regulatory network involving *H*. *pylori*, GECs, ARRDC3, PAR1, and neutrophils, which collectively exert a proinflammatory effect within the gastric microenvironment. Efforts to inhibit this ARRDC3-dependent pathway may provide valuable strategies in treating of *H*. *pylori*–associated gastritis.

## Abstract 2

A regulatory network including H. pylori, GECs, ARRDC3, PAR1, and neutrophils collectively exerts a pro-inflammatory effect within the gastric microenvironment.

## Graphical abstract

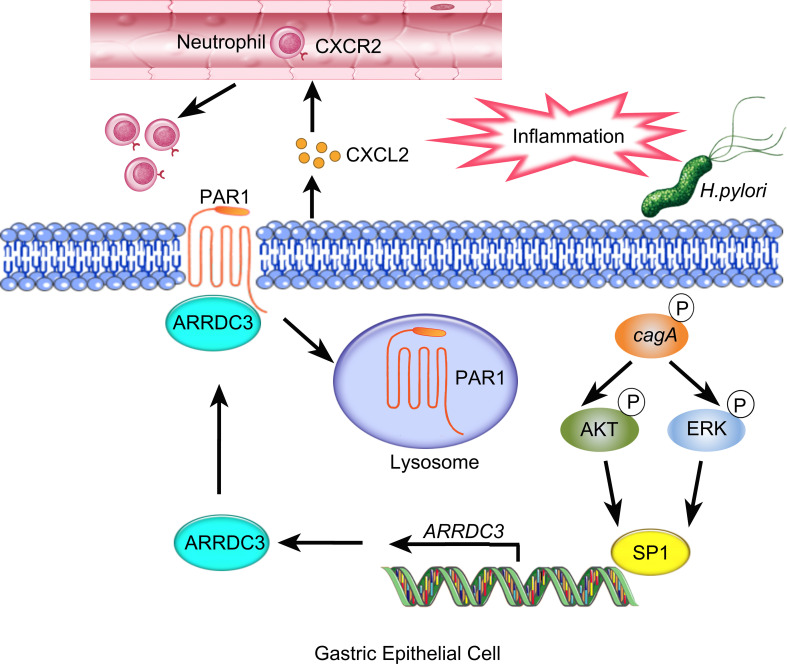

## Introduction

*Helicobacter pylori* is a gram-negative bacterium that infects nearly half of the world’s population as gastric pathogen (1). Persistent *H*. *pylori* infection is a key etiological factor in chronic gastritis and peptic ulcer (2). It was also considered the first class of carcinogenic factors for gastric adenocarcinoma by WHO. However, the mechanisms of developing *H*. *pylori*–associated gastritis remain unclear. The interaction between bacteria and gastric epithelial cells (GECs) might be a key determinant, implied especially by the critical roles of the bacterial virulence factor cytotoxin associated gene A (*cagA*) protein (3, 4).

Arrestins are a family of intracellular proteins that bind to phosphorylated GPCRs and regulate their signal transduction. They are further divided into 3 subfamilies, visual arrestins, β-arrestins, and α-arrestins. Visual arrestins regulate light transduction in photoreceptor cells; like nonvisual arrestins, they are widely distributed and can bind to a wide range of GPCRs to play diverse roles. Importantly, in different inflammatory diseases, arrestins could play opposite roles. For example, β-arrestin 2 promotes inflammation in allergic asthma (5) and inflammatory bowel disease (6), but it plays an antiinflammation role in experimental autoimmune encephalomyelitis (7), rheumatoid arthritis (8), and sepsis (9). Arrestin domain containing 3 (ARRDC3) is an α-arrestin, which was first reported in 2006 as a regulator in PPARγ signaling (10). Recent reports have found that ARRDC3 can regulate GPCR degradation and trafficking (11–13) to participate in various physiological and pathological processes. However, virtually nothing is known about its expression, regulation, and function in *H*. *pylori*–associated gastritis.

In the present study, we have demonstrated that *H*. *pylori–*infected patients and mice show high ARRDC3 expression in GECs. Increased ARRDC3 was induced by *H*. *pylori* *cagA* through the ERK and PI3K-AKT pathways. We further demonstrate that ARRDC3 promotes CXCL2 production through degradation of protease-activated receptor 1 (PAR1), which in turn recruits neutrophils that contribute to inflammation. Collectively, these data point out a potentially novel proinflammation role of ARRDC3 in the GECs during *H*. *pylori*–associated gastritis.

## Results

### ARRDC3 is increased in gastric mucosa of H. pylori–infected patients and mice.

**Figure 1:**
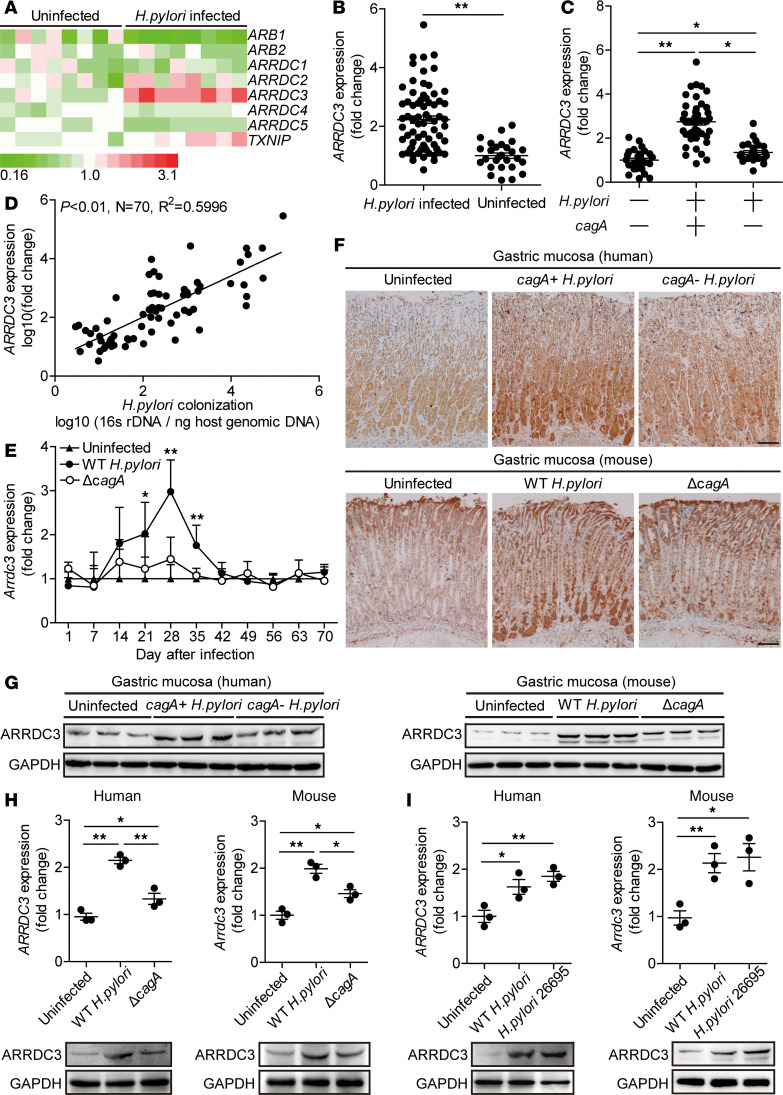
ARRDC3 is increased in gastric mucosa of *H. pylori*–infected patients and mice. (**A**) The mRNA expression profiles of arrestin family members in human primary gastric mucosa of *H*. *pylori*–infected patients (*n* = 8) and uninfected donors (*n* = 8) was analyzed by real-time PCR. (**B**) *ARRDC3* expression in gastric mucosa of *H*. *pylori*–infected (*n* = 70) and uninfected donors (*n* = 27) was compared. (**C**) *ARRDC3* expression in gastric mucosa of *cagA*^+^ *H*. *pylori*–infected (*n* = 44), *cagA*^–^ *H*. *pylori*–infected (*n* = 26), and uninfected donors (*n* = 27) was compared. (**D**) The correlation between *ARRDC3* expression and *H*. *pylori* colonization in gastric mucosa of *H*. *pylori*–infected patients was analyzed. (**E**) Dynamic changes of *Arrdc3* expression in gastric mucosa of WT *H*. *pylori*–infected, *ΔcagA*-infected, and uninfected mice. *n* = 5 per group per time. (**F** and **G**) ARRDC3 protein in gastric mucosa of *cagA*^+^ *H*. *pylori*–infected, *cagA*^–^ *H*. *pylori*–infected, and uninfected donors or in gastric mucosa of WT *H*. *pylori*–infected, *ΔcagA*-infected, and uninfected mice on day 28 p.i. was analyzed by immunohistochemical staining (**F**) and Western blot (**G**). Scale bars: 100 μm. (**H**) *ARRDC3*/*Arrdc3* expression in human primary gastric mucosa from uninfected donors/mice infected with WT *H*. *pylori* or *ΔcagA* ex vivo analyzed by real-time PCR and Western blot (*n* = 3). (**I**) *ARRDC3*/*Arrdc3* expression in human primary gastric mucosa from uninfected donors/mice infected with WT *H*. *pylori* or *H*. *pylori* 26695 ex vivo analyzed by real-time PCR and Western blot (*n* = 3). Data are representative of 2 independent experiments. Data are mean ± SEM and analyzed by Student *t* test, Mann-Whitney *U* test, and 1-way ANOVA. Western blot results are run in parallel and contemporaneously. *P < 0.05, **P < 0.01 for groups connected by horizontal lines or compared with uninfected mice.

o evaluate the potential role of ARRDC3 in *H*. *pylori* infection, we first compared the levels of arrestin family members expressed in human primary gastric mucosa of *H*. *pylori*–infected and uninfected donors. Among them, ARRDC3 was the most increased in gastric mucosa infected by *H*. *pylori* compared with paired uninfected counterparts (Figure 1A). We then confirmed that, compared with uninfected donors, ARRDC3 expression was higher in gastric mucosa of *H*. *pylori*–infected patients (Figure 1B). Furthermore, we demonstrated that, in gastric mucosa of *H*. *pylori*–infected patients, ARRDC3 expression was positively correlated with *H*. *pylori* colonization (Figure 1D), suggesting that *H*. *pylori* induces ARRDC3 expression.

As *cagA* is strongly associated with the development of gastritis (14), we next investigated the relationship between *cagA* and ARRDC3 and found that ARRDC3 expression in *cagA*-positive patients was significantly higher than that in *cagA*-negative individuals (Figure 1C). Consistent with our findings in humans, ARRDC3 expression was also detected in WT *H*. *pylori*–infected but not in *ΔcagA*-infected mice, reaching a peak 28 postinfection (p.i.) days (Figure 1E), indicating a key role for *cagA* to induce ARRDC3 expression during *H*. *pylori* infection in vivo. Furthermore, immunohistochemical staining (Figure 1F) and Western blot analysis (Figure 1G) also showed that the level of ARRDC3 protein was higher in gastric mucosa of *cagA*-positive *H*. *pylori*–infected patients and WT *H*. *pylori*–infected mice, compared with either uninfected or *cagA*-negative patients and *ΔcagA*-infected counterparts, respectively. Similar observations were made when analyzing ARRDC3 protein by immunofluorescence staining (Supplemental Figure 1; supplemental material available online with this article; https://doi.org/10.1172/jci.insight.135849DS1). Furthermore, ARRDC3 mRNA and protein expression increased significantly in human primary gastric mucosa infected with WT *H*. *pylori* ex vivo, compared with that in the samples either not infected or infected with *ΔcagA* (Figure 1H). Infection of human primary gastric mucosa with *H*. *pylori* 26695 (Figure 1I) also increased ARRDC3 expression. Collectively, these data demonstrate that ARRDC3 expression is increased in *H*. *pylori*–infected gastric mucosa of patients and mice.

### GECs infected by H. pylori express ARRDC3.

**Figure 2:**
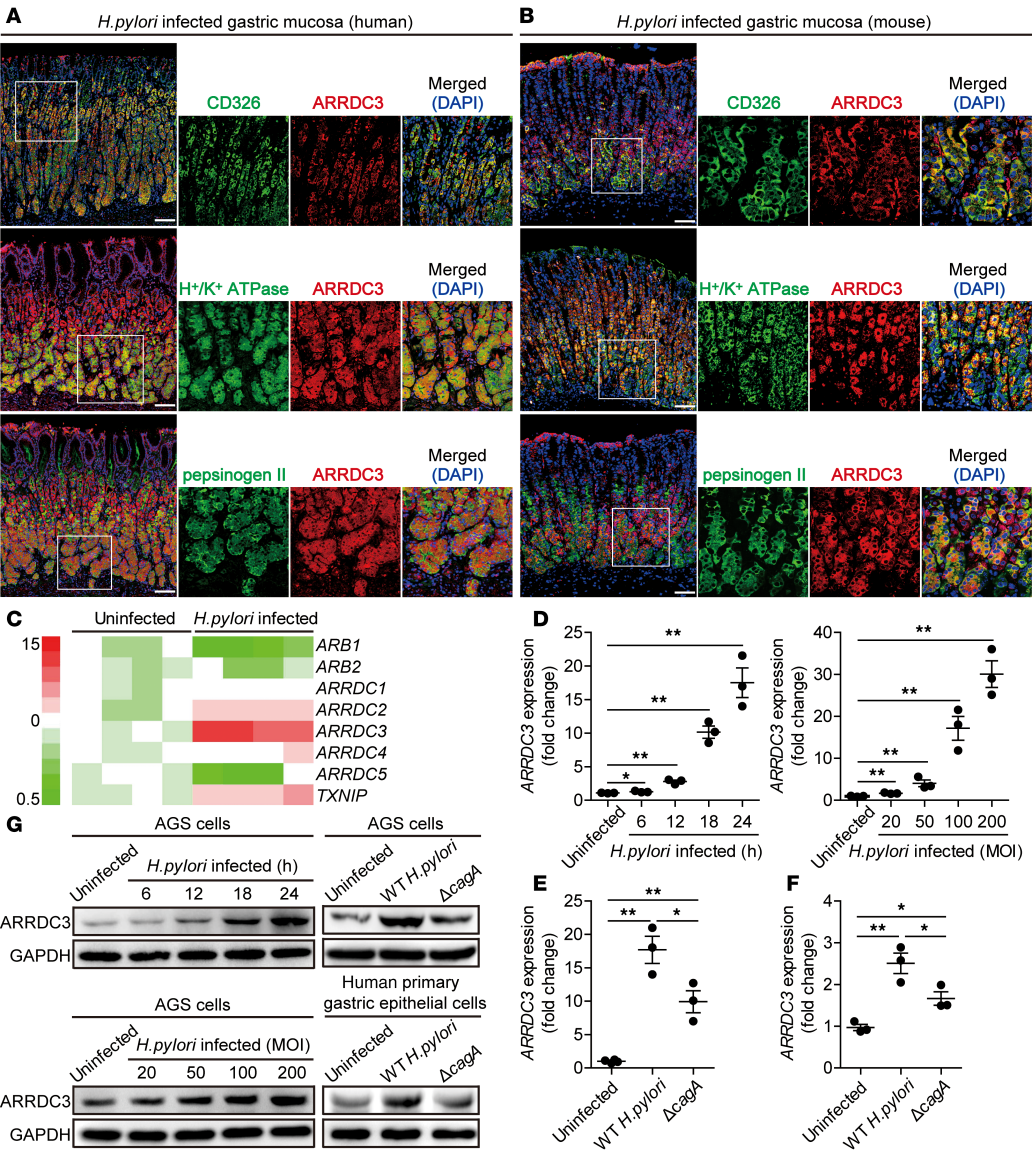
*H. pylori* stimulates gastric epithelial cells to express ARRDC3. (**A** and **B**) Representative immunofluorescence staining images showing ARRDC3-expressing (red) CD326^+^ (green) gastric epithelial cells (GECs), ARRDC3-expressing (red) H^+^/K^+^ ATPase^+^ (green) parietal cells, and ARRDC3-expressing (red) pepsinogen II^+^ chief cells (green) in gastric mucosa of *H*. *pylori*–infected patients (**A**) or *H*. *pylori*–infected mice (**B**). Scale bars: 100 μm. (**C**) The expression of arrestin family members in WT *H*. *pylori*–infected and uninfected AGS cells (MOI = 100, 6 hours) was analyzed by real-time PCR (*n* = 4). (**D** and **G**) *ARRDC3* expression and ARRDC3 protein in WT *H*. *pylori*–infected and uninfected AGS cells at different time points (MOI = 100) or with different MOI (24 hours) were analyzed by real-time PCR and Western blot (*n* = 3). (**E–G**) *ARRDC3* expression and ARRDC3 protein in WT *H*. *pylori*–infected, *ΔcagA*-infected, and uninfected AGS cells (**E** and **G**) and human primary gastric epithelial cells (**F** and **G**) (MOI = 100, 24 hours) were analyzed by real-time PCR and Western blot (*n* = 3). Data are representative of 2 independent experiments. Data are mean ± SEM and analyzed by 1-way ANOVA. Western blot results are run in parallel and contemporaneously. *P < 0.05, **P < 0.01 for groups connected by horizontal lines.

GECs are known to be the first-contacted cell type in gastric mucosa during *H*. *pylori* infection (14). Interestingly, within gastric mucosa of *H*. *pylori*–infected donors or mice, ARRDC3 was expressed in CD326^+^ GECs as well as in H^+^/K^+^ ATPase^+^ parietal cells and pepsinogen II^+^ chief cells (Figure 2, A and B), suggesting that GECs express ARRDC3 in gastric mucosa during *H*. *pylori* infection.

Next, we screened members of arrestin family in AGS cells, a human GEC line, and found that ARRDC3 was the most increased arrestin induced by *H*. *pylori* infection (Figure 2C). We further demonstrated that *H*. *pylori*–infected AGS cells increased ARRDC3 expression in a time-dependent and infection dose–dependent manner (Figure 2, D and G). Notably, compared with uninfected cells or the cells infected with *ΔcagA*, WT *H*. *pylori*–infected AGS cells (Figure 2, E and G) and human primary GECs (Figure 2, F and G) also potently increased ARRDC3 expression. Similar observations were made when using other human GEC lines (Supplemental Figure 2, A and B). Furthermore, infection of human GEC lines with WT *H. pylori* or *H. pylori* 26695 (Supplemental Figure 2, D–F) also increased ARRDC3 expression. also increased ARRDC3 expression. To explore the mechanism of ARRDC3 induction by *H. pylori*, we performed transwell assays and found that bacterium-cell contact was necessary for the induction of ARRDC3 expression in AGS cells infected with WT *H. pylori* or *H. pylori* 26695 (Supplemental Figure 2C). Taken together, our data demonstrate that *H*. *pylori* induces ARRDC3 expression in GECs.

### H. pylori induces GEC to express ARRDC3 via ERK and PI3K-AKT pathways.

**Figure 3:**
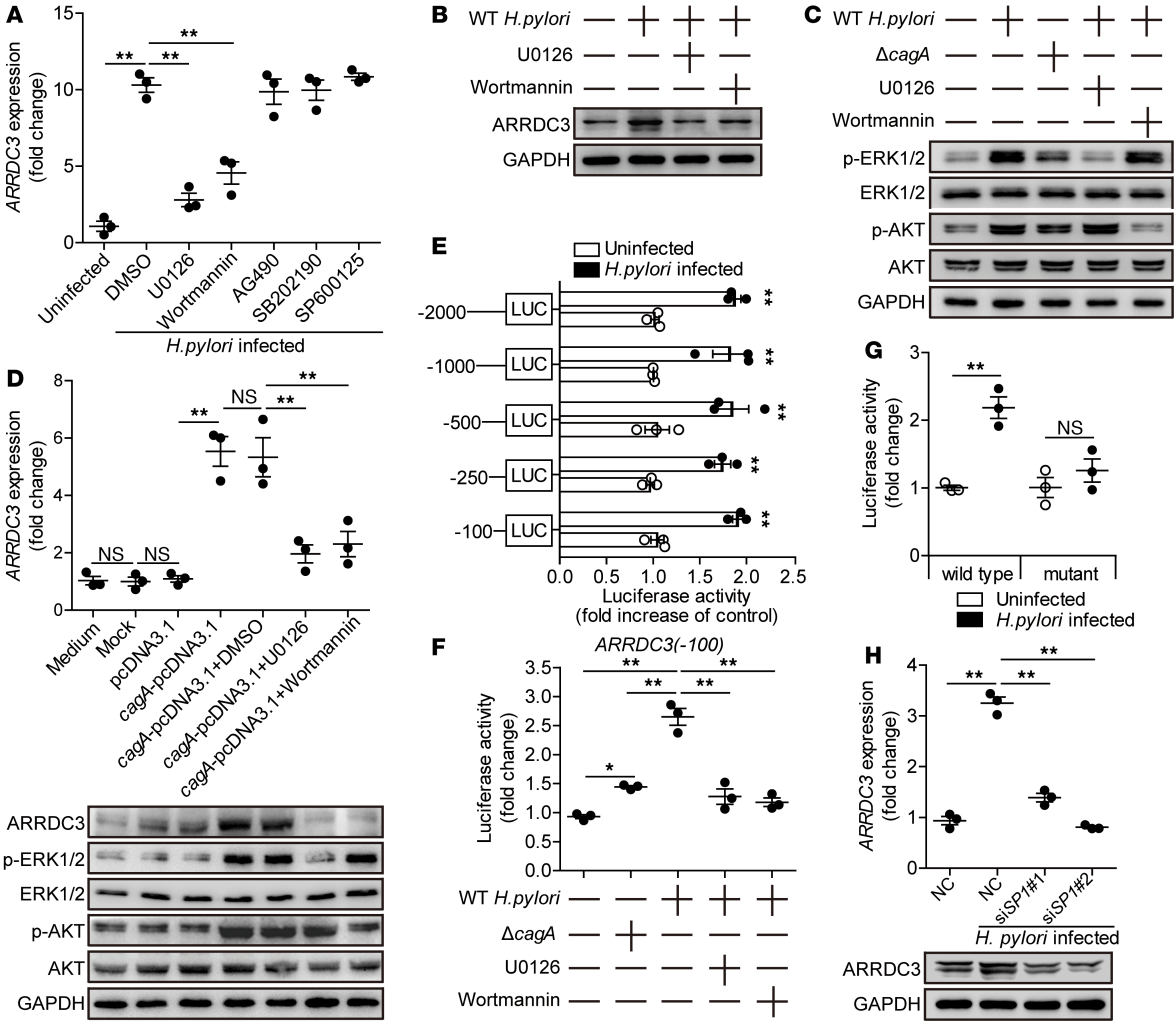
*H. pylori* induces gastric epithelial cells to express ARRDC3 via ERK and PI3K-AKT pathways. (**A**) AGS cells were pretreated with signal pathway inhibitors and then infected with WT *H*. *pylori* (MOI = 100) for 24 hours. *ARRDC3* expression in AGS cells was compared (*n* = 3). (**B**) AGS cells were pretreated with U0126 (an ERK inhibitor) or Wortmannin (a PI3K-AKT inhibitor) and then infected with WT *H*. *pylori* (MOI = 100) for 24 hours. ARRDC3 protein was analyzed by Western blot. (**C**) AGS cells were pretreated with U0126 (an ERK inhibitor) or Wortmannin (a PI3K-AKT inhibitor) and then infected with WT *H*. *pylori* or *ΔcagA* (MOI = 100) for 24 hours. ERK1/2, p-ERK1/2, AKT, and p-AKT proteins were analyzed by Western blot. (**D**) AGS cells were transfected with plasmids pcDNA3.1 or *cagA*-pcDNA3.1 for 24 hours and then treated with or without U0126 or Wortmannin for 2 hours before being cultured for an additional 24 hours. *ARRDC3* expression and ARRDC3 protein were analyzed by real-time PCR and Western blot (*n* = 3). (**E**) AGS were transfected with luciferase reporter constructs containing the *ARRDC3*-luc promoter for 4 hours. Luciferase activity was measured to assess promoter activity after WT *H*. *pylori* infection (MOI = 100) for 24 hours. (**F**) AGS were transfected with luciferase reporter constructs containing the *ARRDC3*-luc promoter for 4 hours. Luciferase activity was measured to assess promoter activity after WT *H*. *pylori* (pretreated with or without U0126 or Wortmannin) or *ΔcagA* infection (MOI = 100) for 24 hours. (**G**) AGS cells were transfected with the *ARRDC3*-luc (–100/0) construct (WT) or a mutant construct (mutant). Luciferase activity was measured to assess promoter activity after WT *H*. *pylori* infection (MOI = 100) for 24 hours. (**H**) *SP1* siRNA (si*SP1* 1 and si*SP1* 2) or nonspecific control siRNA (NC) pretreated AGS cells were infected with WT *H*. *pylori* (MOI = 100) for 24 hours. *ARRDC3* expression were analyzed by real-time PCR and Western blot (*n* = 3). Data are representative of 2 independent experiments. Data are mean ± SEM and analyzed by Student *t* test, Mann-Whitney *U* test, and 1-way ANOVA. Western blot results are run in parallel and contemporaneously. *P < 0.05, **P < 0.01 for groups connected by horizontal lines or compared with uninfected cells.

To explore the underlying mechanism of ARRDC3 induction in GECs by *H*. *pylori*, we performed signaling pathways blocking experiments, and the results show that only blocking the signal transduction of ERK or PI3K-AKT pathway with inhibitor U0126 or Wortmannin effectively decreased ARRDC3 expression (Figure 3, A and B). Furthermore, ERK1/2 and AKT, direct ERK and PI3K-AKT pathway downstream substrates, were predominantly phosphorylated in AGS cells after being infected with *H*. *pylori*, and this was more noticeable when infected with a WT *H*. *pylori* compared with *ΔcagA*, which was abolished when pretreated with inhibitor U0126 or Wortmannin (Figure 3C). To demonstrate that *cagA* is essential and sufficient to induce ARRDC3 expression, we transiently expressed *cagA* in AGS cells in the presence or absence of U0126 or Wortmannin; the results mirrored those from *H*. *pylori* infection (Figure 3D). To further investigate how *H*. *pylori* induces *ARRDC3* gene transcription, we constructed a series of *ARRDC3*-luc promoter constructs of varying lengths (–2000/0, –1000/0, –500/0, –250/0, –100/0) and performed luciferase reporter assay, and the results show that *ARRDC3* promoter (–100/0) mediated transcription responsiveness to *H*. *pylori* (Figure 3E). Furthermore, compared with Δ*cagA* infection, infection with WT *H*. *pylori* significantly enhanced this luciferase activity, and pretreatment with inhibitor U0126 or Wortmannin abrogated the increase in luciferase activity induced by WT *H*. *pylori* (Figure 3F).

The PROMO tool V.8.3 of TRANSFAC showed that *ARRDC3* promoter (–100/0) contains a Specificity protein 1 (SP1) binding site (comprising the sequence AGGGCGGACA). To functionally confirm the role of this binding site in driving *H*. *pylori*–mediated *ARRDC3* gene transcription, *ARRDC3*-luc promoter constructs with a mutation in the binding site element were assessed for cellular luciferase activity. In agreement with our hypothesis, luciferase activity was abrogated with a site-directed mutant promoter construct (Figure 3G). Next, SP1 was suppressed by siRNA (Supplemental Figure 3), and then *H*. *pylori–*induced ARRDC3 expression was abrogated following SP1 suppression (Figure 3H). Taken together, these findings clearly demonstrate that *H*. *pylori* induces ARRDC3 expression in GECs via activating ERK and PI3K-AKT signaling pathways.

### ARRDC3 has proinflammatory effects during H. pylori infection.

**Figure 4:**
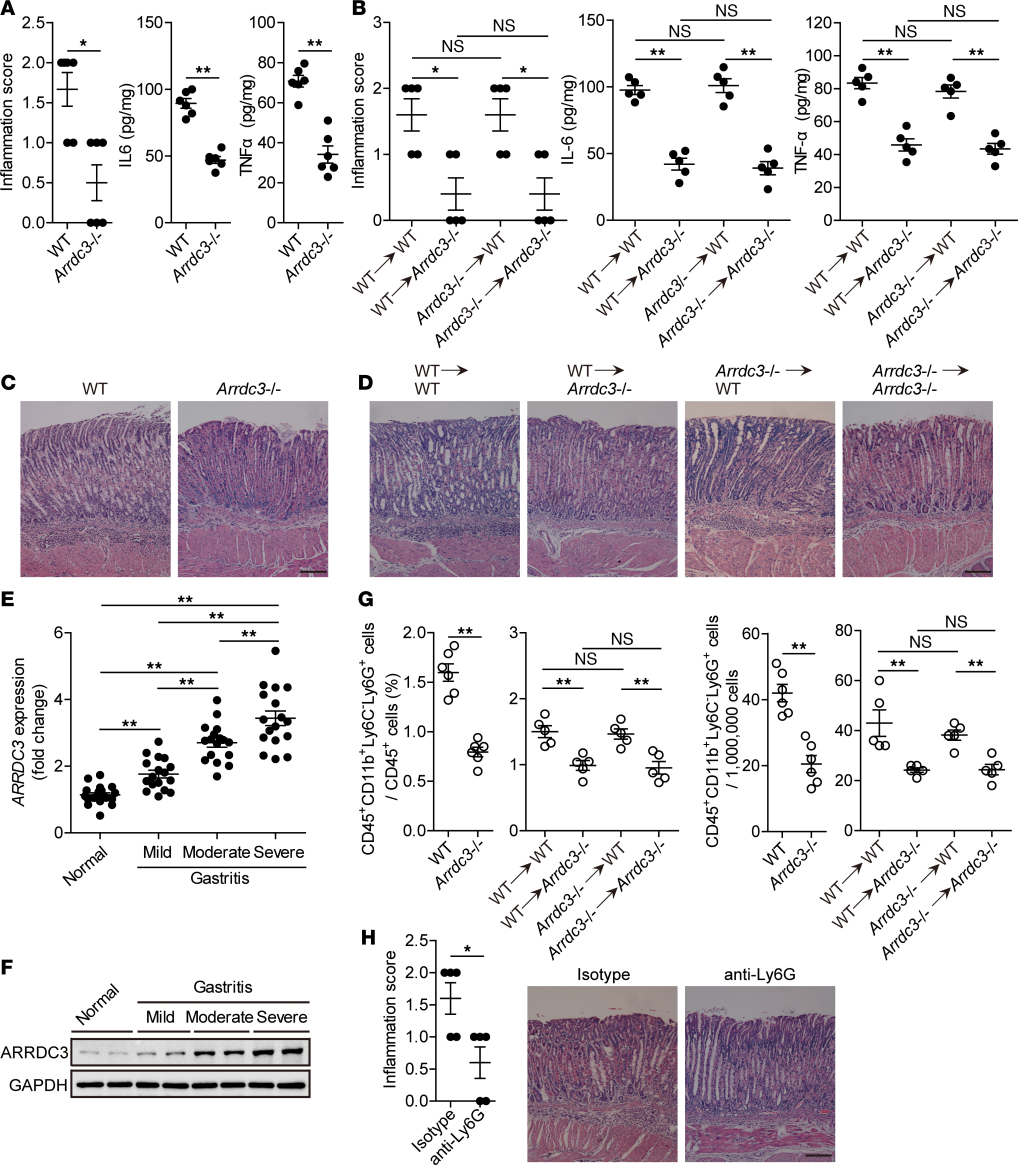
ARRDC3 has proinflammatory effects during *H. pylori* infection. (**A** and **B**) Histological scores of inflammation and IL-6 and TNF-α protein in gastric mucosa of WT *H*. *pylori*–infected WT and *Arrdc3*^–/–^ mice (*n* = 6) (**A**) or in gastric mucosa of WT *H*. *pylori*–infected BM chimera mice (*n* = 5) (**B**) on day 28 p.i. were compared. (**C** and **D**) Representative H&E staining images showed inflammation in gastric antra of WT *H*. *pylori*–infected WT and *Arrdc3*^–/–^ mice (**C**) or in gastric antra of WT *H*. *pylori*–infected BM chimera mice (**D**) on day 28 p.i. Scale bars: 100 μm. (**E** and **F**) *ARRDC3* expression (**E**) and ARRDC3 protein (**F**) in gastric mucosa of *H*. *pylori*–infected patients with mild (*n* = 17), moderate (*n* = 17), severe inflammation (*n* = 17), and uninfected donors with normal gastric histopathology (*n* = 19) was compared. (**G**) CD45^+^CD11b^+^Ly6C^–^Ly6G^+^ neutrophil levels in gastric mucosa of WT *H*. *pylori*–infected WT and *Arrdc3*^–/–^ mice (*n* = 6) or in gastric mucosa of WT *H*. *pylori*–infected BM chimera mice (*n* = 5) on day 28 p.i. were compared. (**H**) Histological scores of inflammation in gastric mucosa of WT *H*. *pylori*–infected mice injected with Abs against Ly6G or corresponding isotype control Abs on day 28 p.i. were compared (*n* = 5). Scale bars: 100 μm. Data are representative of 2 independent experiments. Data are mean ± SEM and analyzed by Student *t* test, Mann-Whitney *U* test, and 1-way ANOVA. Western blot results are run in parallel and contemporaneously. *P < 0.05, **P < 0.01 for groups connected by horizontal lines.

To evaluate the possible biological effects of ARRDC3 in *H*. *pylori*–associated gastritis in vivo, we evaluated the inflammatory response in gastric mucosa on day 28 p.i. Compared with WT mice, Arrdc3^–/–^ mice showed significantly less inflammation (Figure 4, A and C) and less expression of proinflammatory cytokines IL-6 and TNF-α in gastric mucosa (Figure 4A and Supplemental Figure 4A). We next generated BM chimera mice and found that non-BM–derived ARRDC3-expressing cells were largely responsible for gastric inflammation (Figure 4, B and D) and less expression of proinflammatory cytokines IL-6 and TNF-α (Figure 4B and Supplemental Figure 4B) during *H. pylori* infection in this model. Furthermore, higher ARRDC expression was strongly associated with more severe gastritis within gastric mucosa of patients infected with *H*. *pylori* (Figure 4E). Collectively, these results suggest that ARRDC3 has effects on promoting inflammation during *H*. *pylori* infection in vivo.

*H. pylori*–associated gastritis is characterized as inflammatory cell infiltration under chronic inflammatory conditions (15). To investigate whether increased ARRDC3 regulated immune cell infiltration into the gastric mucosa during *H*. *pylori* infection, we compared the levels of CD45^+^CD11b^+^Ly6C^–^Ly6G^+^ neutrophils, CD45^+^CD11b^+^Ly6G^–^Ly6C^+^ monocytes, CD45^+^CD3^+^ T cells, CD45^+^CD3^+^CD8^–^CD4^+^ T cells, and CD45^+^CD3^+^CD4^–^CD8^+^ T cells in gastric mucosa on day 28 p.i. between WT and *Arrdc3^–/–^* mice, and we found that abolishing ARRDC3 in *Arrdc3^–/–^* mice only reduced gastric but not blood or BM CD45^+^CD11b^+^Ly6C^–^Ly6G^+^ neutrophils (Figure 4F and Supplemental Figure 5 and 6). These results were also confirmed by our BM chimera experiments in which non-BM–derived ARRDC3-expressing cells were largely responsible for the neutrophil accumulation in gastric mucosa during *H*. *pylori* infection (Figure 4F and Supplemental Figure 5 and 6). Finally, we determined the contribution of neutrophils to *H*. *pylori*–induced inflammation by Ab-mediated neutrophil depletion. Neutrophil depletion was verified by flow cytometry (Figure 4G), and neutropenic mice showed less inflammation than control mice (Figure 4H). Taken together, our data demonstrate that ARRDC3 plays an essential role in neutrophil accumulation, which contributes to inflammation in gastric mucosa during *H*. *pylori* infection.

### ARRDC3 promotes neutrophil accumulation in gastric mucosa in vivo and migration in vitro during H. pylori infection via CXCL2.

**Figure 5:**
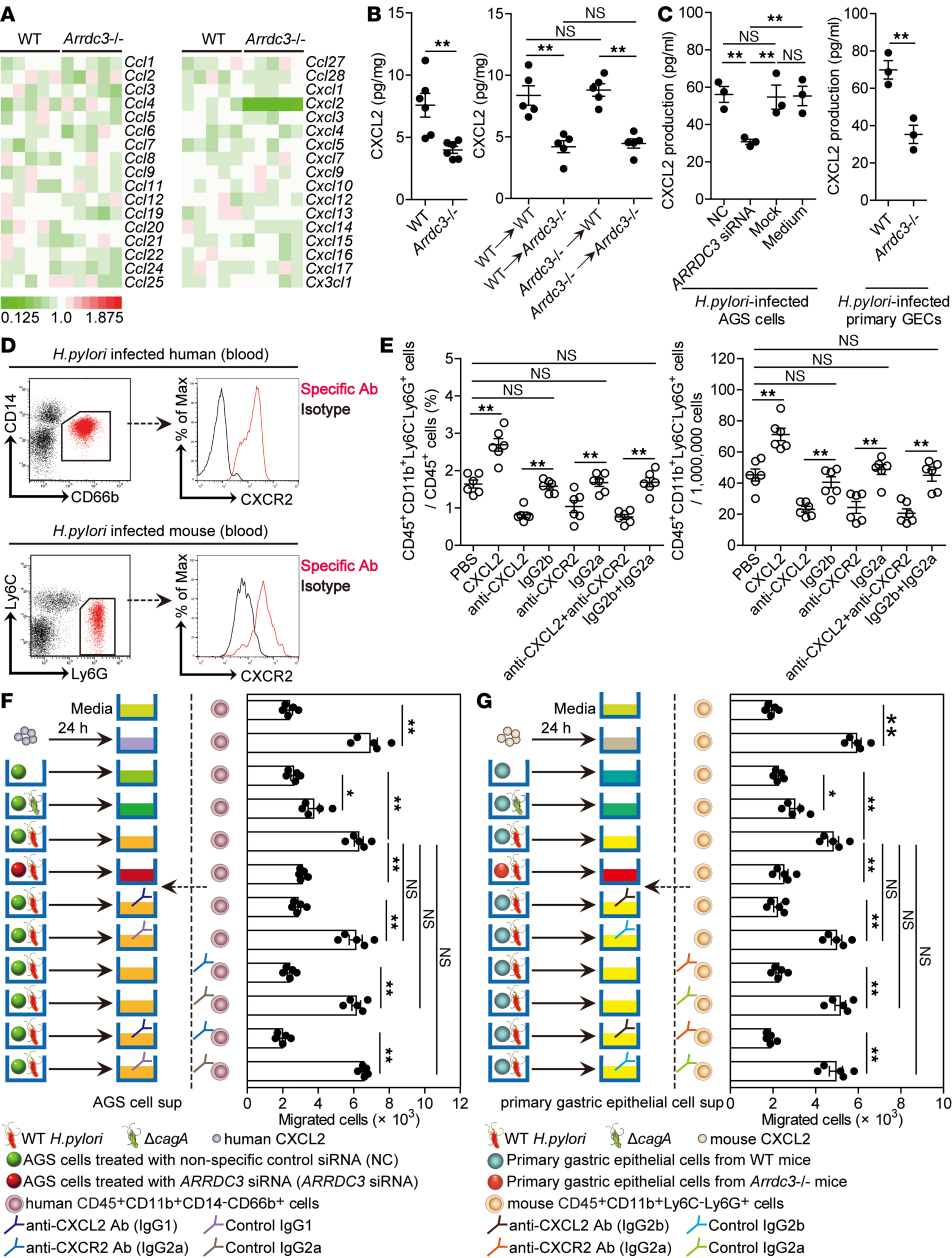
ARRDC3 promotes neutrophil accumulation in gastric mucosa in vivo and migration in vitro during *H. pylori* infection via CXCL2. (**A**) The expression of chemokine family members in gastric mucosa of WT *H*. *pylori*–infected WT and *Arrdc3*^–/–^ mice on day 28 p.i. was analyzed by real-time PCR (*n* = 5). (**B**) CXCL2 expression in gastric mucosa of WT *H*. *pylori*–infected WT and *Arrdc3*^–/–^ mice (*n* = 6) or in gastric mucosa of WT *H*. *pylori*–infected BM chimera mice (*n* = 5) on day 28 p.i. was compared. (**C**) *ARRDC3* siRNA, nonspecific control siRNA (NC), or lipo3000 only (mock) pretreated AGS cells or AGS cells without treatment (medium) and primary gastric epithelial cells (GECs) from uninfected WT and *Arrdc3*^–/–^ mice were infected with WT *H*. *pylori* (MOI = 100) for 24 hours. CXCL2 production was measured in cell culture supernatants by ELISA (*n* = 3). (**D**) CXCR2 expression on human CD45^+^CD11b^+^CD14^–^CD66b^+^ neutrophils in blood of *H*. *pylori*–infected patients or on mouse CD45^+^CD11b^+^Ly6C^–^Ly6G^+^ neutrophils in blood of WT *H*. *pylori*–infected mice on day 28 p.i. (**E**) CD45^+^CD11b^+^Ly6C^–^Ly6G^+^ neutrophil levels in gastric mucosa of WT *H*. *pylori*–infected mice injected with CXCL2 or PBS control, or Abs against CXCL2 and/or Abs against CXCR2 or corresponding isotype control Abs on day 28 p.i. were compared (*n* = 5). (**F** and **G**) Human CD45^+^CD11b^+^CD14^–^CD66b^+^ neutrophil migration (**F**) and mouse CD45^+^CD11b^+^Ly6C^–^Ly6G^+^ neutrophil migration (**G**) was assessed by transwell assays as described in Methods and statistically analyzed (*n* = 5). Data are representative of 2 independent experiments. Data are mean ± SEM and analyzed by Student *t* test, Mann-Whitney *U* test, and 1-way ANOVA. *P < 0.05, **P < 0.01 for groups connected by horizontal lines. sup, supernatant.

Chemotaxis plays important roles in neutrophil migration (16). We were therefore interested to know if ARRDC3 regulated chemokine production in gastric mucosa. We screened chemokines in gastric mucosa on day 28 p.i. between WT and *Arrdc3^–/–^* mice and found that only CXCL2 expression was reduced in *Arrdc3^–/–^* mice (Figure 5, A and B, and Supplemental Figure 7A). Again, BM chimera experiments confirmed that non-BM–derived ARRDC3-expressing cells were largely responsible for CXCL2 expression in gastric mucosa during *H*. *pylori* infection (Figure 5B and Supplemental Figure 7A). Taken together, our data demonstrate that ARRDC3 plays an essential role in inducing CXCL2 production in gastric mucosa during *H*. *pylori* infection.

Next, we tried to determine whether neutrophil accumulation during *H*. *pylori* infection was regulated by the ARRDC3-CXCL2 axis. To begin, we found that CXCL2 production from AGS cells, as well as from mouse primary GECs, was regulated in a ARRDC3-dependent manner (Figure 5C and Supplemental Figure 7B). Then, blood neutrophils collected from *H*. *pylori*–infected patients and mice expressed CXCR2, the chemokine receptor of CXCL2 (Figure 5D). Finally, we conducted a series of loss- and gain-of-function experiments in vivo involving CXCL2 and/or CXCR2, and we evaluated neutrophil response in gastric mucosa on day 28 p.i. CXCL2 administration significantly increased neutrophil accumulation; conversely, neutralization of CXCL2 and/or CXCR2 significantly reduced neutrophil accumulation (Figure 5E).

To further evaluate the contribution of an ARRDC3-CXCL2 axis to neutrophil migration in vitro, human CD45^+^CD11b^+^CD14^–^CD66b^+^ neutrophil chemotaxis assay was performed and demonstrated that culture supernatants from WT *H*. *pylori*–infected AGS cells pretreated with nonspecific control siRNA (NC) induced significantly more neutrophil migration than the supernatants collected from WT *H*. *pylori*–infected AGS cells pretreated with *ARRDC3* siRNA or that from *ΔcagA*-infected AGS cells pretreated with NC; this effect was lost upon pretreatment with neutralizing Abs against CXCL2 and/or CXCR2 (Figure 5F). However, there is no significant difference of CXCR2 expression on human blood CD45^+^CD11b^+^CD14^–^CD66b^+^ neutrophils between *H. pylori*–infected patients and uninfected donors (Supplemental Figures 7C).

Similarly, culture supernatant collected from WT *H*. *pylori*–infected primary GECs of WT mice also induced significantly more mouse CD45^+^CD11b^+^Ly6C^–^Ly6G^+^ neutrophil migration than those from WT *H*. *pylori*–infected primary GECs of *Arrdc3^–/–^* mice or those from *ΔcagA*-infected primary GECs of WT mice; this effect was also lost upon pretreatment with neutralizing Abs against CXCL2 and/or CXCR2 (Figure 5G). Collectively, these results suggest that an ARRDC3-CXCL2 axis contributes to neutrophil accumulation within gastric mucosa during *H*. *pylori* infection, which may contribute to gastritis.

### ARRDC3 exerts proinflammatory effects via downregulating PAR1 during H.

**Figure 6:**
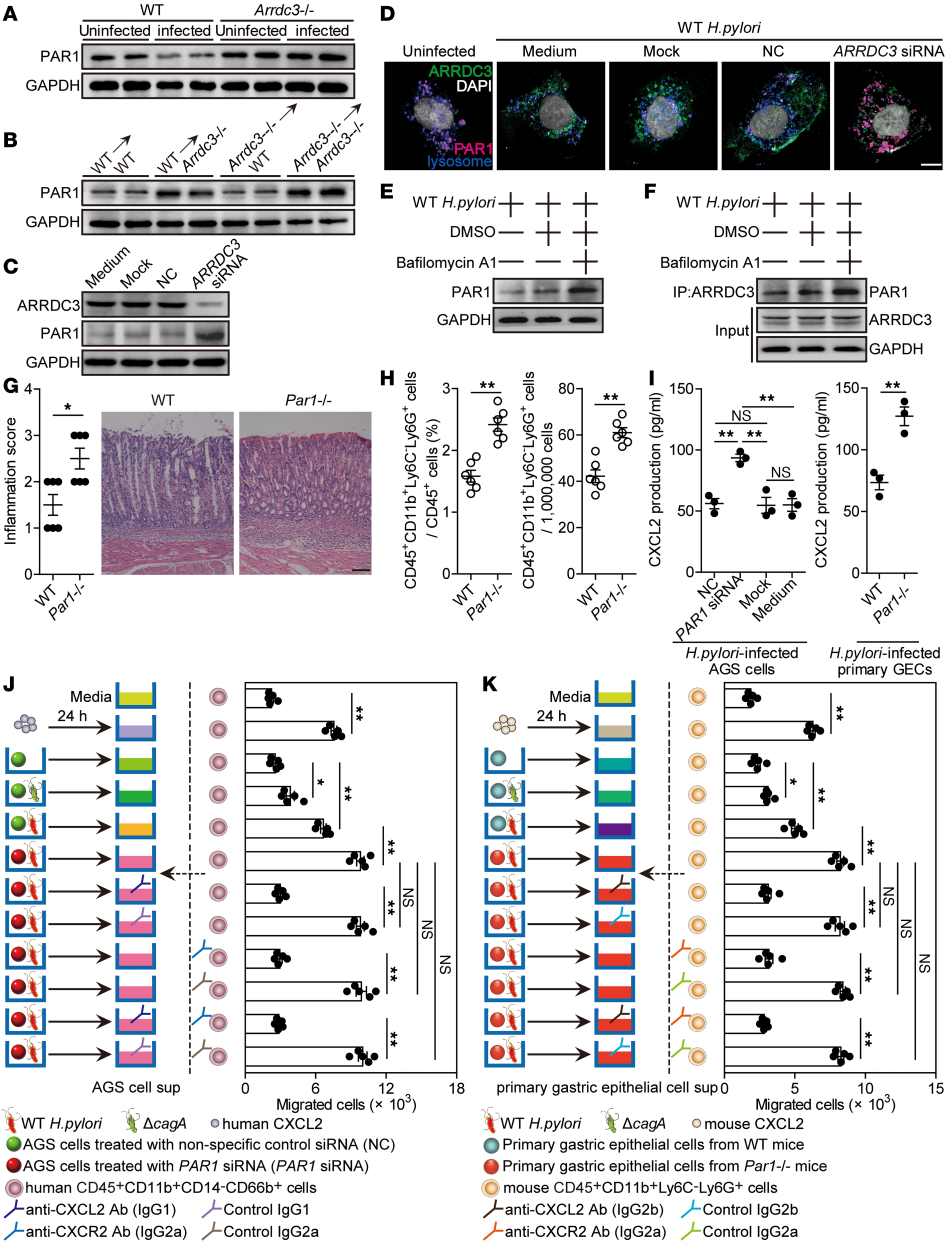
ARRDC3 exerts proinflammatory effects via downregulating PAR1 during *H. pylori* infection. (**A**) PAR1 expression in gastric mucosa of uninfected or WT *H*. *pylori*–infected WT and *Arrdc3*^–/–^ mice on day 28 p.i. was analyzed by Western blot. (**B**) PAR1 expression in gastric mucosa of WT *H*. *pylori*–infected BM chimera mice on day 28 p.i. was analyzed by Western blot. (**C** and **D**) *ARRDC3* siRNA, nonspecific control siRNA (NC), or lipo3000 only (mock) pretreated AGS cells or AGS cells without treatment (medium) were infected with WT *H*. *pylori* (MOI = 100) for 24 hours. ARRDC3 and PAR1 expression was analyzed by Western blot (**C**) or immunofluorescence staining (**D**). Scale bar: 1 μm. (**E** and **F**) ARRDC3 was immunoprecipitated from AGS cells infected with WT *H*. *pylori* (MOI = 100, 24 hours) pretreated with or without Bafilomycin A1. PAR1 expression was analyzed by Western blot (**E**). Interacting PAR1 and precipitated ARRDC3 in the immunocomplexes were further analyzed by Western blotting using anti-ARRDC3 and anti-PAR1. Normal rabbit IgG was used as a negative control (**F**). (**G** and **H**) Histological scores of inflammation (**G**) and CD45^+^CD11b^+^Ly6C^–^Ly6G^+^ neutrophil level (**H**) in gastric mucosa of WT *H*. *pylori*–infected WT and *Par1*^–/–^ mice on day 28 p.i. were compared (*n* = 6). Representative H&E staining images showed inflammation in gastric antra of WT *H*. *pylori*–infected WT and *Par1*^–/–^ mice on day 28 p.i. Scale bars: 100 μm. (**I**) *PAR1* siRNA, nonspecific control siRNA (NC), or lipo3000 only (mock) pretreated AGS cells or AGS cells without treatment (medium), and primary gastric epithelial cells (GECs) from uninfected WT and *Par1*^–/–^ mice were infected with WT *H*. *pylori* (MOI = 100) for 24 hours. CXCL2 production was measured in cell culture supernatants by ELISA (*n* = 3). (**J** and **K**) Human CD45^+^CD11b^+^CD14^–^CD66b^+^ neutrophil migration (**J**) and mouse CD45^+^CD11b^+^Ly6C^–^Ly6G^+^ neutrophil migration (**K**) was assessed by transwell assays as described in Methods and statistically analyzed (*n* = 5). Data are representative of 2 independent experiments. Data are mean ± SEM and analyzed by Student *t* test, Mann-Whitney *U* test, and 1-way ANOVA. Western blot results are run in parallel and contemporaneously. *P < 0.05, **P < 0.01, for groups connected by horizontal lines. sup, supernatant.

*pylori infection*. It has previously been shown that PAR1, a GPCR, is regulated via ARRDC3-mediated lysosomal degradation in breast carcinoma (17). To see whether a similar mechanism might operate in the function of ARRDC3, we first quantified the PAR1 levels in gastric mucosa of uninfected or WT *H*. *pylori*–infected WT and *Arrdc3*^–/–^ mice on day 28 p.i., and we found that PAR1 in WT mice on day 28 p.i. was significantly decreased compared with that in uninfected WT mice, whereas there was no change of PAR1 between uninfected *Arrdc3^–/–^* mice and *Arrdc3^–/–^* mice on day 28 p.i. (Figure 6A). Further comparing the PAR1 levels in gastric mucosa of WT and *Arrdc3*^–/–^ mice and their BM chimera mice on day 28 p.i., we found that PAR1 in *Arrdc3*^–/–^ mice was substantially increased when compared with that in WT mice (Figure 6A) and that non-BM–derived ARRDC3-expressing cells were largely responsible for a PAR1 increase in gastric mucosa during *H*. *pylori* infection (Figure 6B). Similar results were obtained in *H*. *pylori*–infected AGS cells pretreated with *ARRDC3* siRNA (Figure 6C). Then, immunofluorescence staining showed increased ARRDC3 coupled with decreased PAR1 in *H*. *pylori*–infected AGS cells, when compared with uninfected AGS cells; such changes were inhibited when AGS cells were pretreated with ARRDC3 siRNA (Figure 6D). Immunofluorescence staining also showed a lysosomal localization of PAR1 in *H*. *pylori*–infected AGS cells (Figure 6D). Next, to see whether lysosome mediated PAR1 degradation, we inhibited lysosomal function with Bafilomycin A1 and found increased PAR1 in *H*. *pylori*–infected AGS cells (Figure 6E). To further address whether ARRDC3-mediated lysosomal degradation led to the decrease of PAR1, we again inhibited lysosomal function with Bafilomycin A1, followed by IP with anti-ARRDC3 in *H*. *pylori*–infected AGS cells. We again found increased ARRDC3-associated PAR1 (Figure 6F). Taken together, our data demonstrate that PAR1 is downregulated via ARRDC3-mediated lysosomal degradation during *H*. *pylori* infection.

To evaluate the possible biological effects of PAR1 in *H*. *pylori*–associated gastritis in vivo, we evaluated the inflammatory response in gastric mucosa on day 28 p.i. Compared with WT mice, *Par1^–/–^* mice showed significantly more inflammation (Figure 6G) and higher CD45^+^CD11b^+^Ly6C^–^Ly6G^+^ neutrophil infiltration (Figure 6H) in gastric mucosa. Then, we found that CXCL2 production from AGS cells, as well as from mouse primary GECs, was negatively regulated in a PAR1-dependent manner (Figure 6I).

To further evaluate the contribution of a PAR1-CXCL2 axis to neutrophil migration in vitro, a human CD45^+^CD11b^+^CD14^–^CD66b^+^ neutrophil chemotaxis assay was performed and demonstrated that culture supernatants from WT *H*. *pylori*–infected AGS cells pretreated with *PAR1* siRNA induced significantly more neutrophil migration than the supernatants collected from WT *H*. *pylori*–infected AGS cells pretreated with NC; this effect was lost upon pretreatment with neutralizing Abs against CXCL2 and/or CXCR2 (Figure 6J).

Similarly, culture supernatant collected from WT *H*. *pylori*–infected primary GECs of *Par1^–/–^* mice also induced significantly more mouse CD45^+^CD11b^+^Ly6C^–^Ly6G^+^ neutrophil migration than those from WT *H*. *pylori*–infected primary GECs of WT mice; this effect was also lost upon pretreatment with neutralizing Abs against CXCL2 and/or CXCR2 (Figure 6K). Collectively, these results suggest that a PAR1-CXCL2 axis contributes to neutrophil accumulation within gastric mucosa during *H*. *pylori* infection, which may contribute to gastritis.

## Discussion

During *H*. *pylori* infection the host mounts an immune response, which may develop into a chronic inflammation leading to damages in gastric mucosa. The severity of inflammation arisen by *H*. *pylori* infection is the key factor that drives progression to its associated chronic gastritis, gastric ulcer, and gastric cancer. Notably, PAR1, a GPCR, is reported as a suppressor of inflammation in *H*. *pylori* infection (18), while the major function of arrestins is to inhibit and degrade GPCR, suggesting a possible role of arrestins in *H*. *pylori*–driven pathological inflammation. We have screened the expression of arrestin family members in human primary gastric mucosa of *H*. *pylori*–infected and uninfected donors, and we found that 2 arrestins (ARRDC3 and TXNIP) are increased and 2 other arrestins (ARB1 and ARRDC5) are decreased. It is previously reported that TXNIP is slightly increased in *H*. *pylori*–infected GECs (19), which may promote inflammation by binding to NLRP3 inflammasome (20). In addition, nonhematopoietic ARB1 has been reported to inhibit inflammation in a murine model of polymicrobial sepsis through inhibiting NF-κB signaling (21). In this study, we demonstrate that *H*. *pylori* infection led to an increase of ARRDC3 in GECs, which may be important in *H*. *pylori*–induced gastritis through degradation of PAR1.

It has been previously reported that ARRDC3 is associated with many diseases by acting on GPCRs — for instance, ARRDC3 can prevent energy expenditure and promote obesity through inhibiting β2-adrenergic receptor in male mice (22), and ARRDC3 also can suppress breast cancer and prostate cancer progression by regulating GPCR sorting or integrin β4 degradation (17, 23, 24). However, to date, the relationship between ARRDC3 and infection or inflammation has not been reported.

When *H*. *pylori* enters the stomach and adheres to GECs, a major bacterial virulence factor *cagA* is injected into the host cells by the type IV secretion system and interferes with several signaling transduction pathways, such as Wnt–β-catenin, PI3K-AKT, and JAK-STAT3 pathways (25), and plays an important role in chronic gastritis and cancer development. The content of *cagA* is variable in different strains. Clinically isolated strains in East Asia possess more *cagA* compared with strains common in Western populations (26) or South African populations (27). This may be one of the reasons for the high prevalence of *H*. *pylori*–related gastric diseases in the East Asian population. Here, in GECs, *cagA*-dependent ERK and PI3K-AKT pathway activation is required for *H*. *pylori*–induced ARRDC3 expression. In addition, we found the transcription factor SP1 binding site in the core promoter regions of ARRDC3 through bioinformatics analysis, and further experiments discovered that transcription factor SP1 may be the downstream molecule of ERK and PI3K-AKT pathway and involved in *H*. *pylori*–induced ARRDC3 upregulation. Our observations are in keeping with findings showing that SP1 can be activated by *H*. *pylori* *cagA* through ERK and PI3K-AKT pathways (28, 29). There is no doubt that virulence factor *cagA* has a crucial role in the *H*. *pylori–*associated diseases. However, the diversity of clinical outcomes after *H*. *pylori* infection indicates the complexity of its pathogenic mechanism. In both our in vitro cell model and in vivo mouse model, our data show a moderate response of the *ΔcagA H*. *pylori* in inducing ARRDC3 expression. Considering that other factors may be involved in the regulation of ARRDC3 expression, further studies of the underlying mechanisms are needed.

PAR1 plays diverse roles in inflammatory diseases, such as increasing ROS production to promote inflammation in bleomycin-induced lung injury (30). Conversely, PAR1 is also reported to protect the host against severe gastritis during *H*. *pylori* infection (18), and its deficiency leads to increased secretion of some cytokines and chemokines in gastric mucosa during *H*. *pylori* infection (18, 31). Previous studies have shown that ARRDC3 facilitates lysosomal degradation of PAR1 by mediating WWP2 interaction with ALIX (12). Here, within GECs, we find ARRDC3 could promote PAR1 lysosomal degradation, which is consistent with the reports of PAR1 as the target of several arrestins (17, 32). Gastritis is frequently characterized by a dense infiltration of neutrophils in the lamina propria, and neutrophils are thought to play a critical role during *H*. *pylori* infection (33). Previous studies have shown the increase of chemokine CXCL2 expression in the gastric mucosa of *H*. *pylori–*infected patients (34), and CXCL2 specifically attracts neutrophils by interacting with CXCR2 (35). In our case, we identified a downstream mechanism for ARRDC3; it promotes GECs to produce CXCL2, which attracts CXCR2-expressing neutrophil migration and leads to gastric inflammation during *H*. *pylori* infection. Further chimera mouse experiments indicate that non-BM–derived ARRDC3 contributes to this inflammation. Moreover, in vivo neutrophil depletion experiments and in vitro neutrophil migration assays verify that *H*. *pylori* increases inflammation characterized by the neutrophil influx via the ARRDC3-PAR1-CXCL2 axis.

Taken together, ARRDC3 expression is induced in GECs by *cagA*-positive *H*. *pylori* strains via ERK and PI3K-AKT pathways and transcription factor SP1 activation. Furthermore, ARRDC3 promotes CXCL2 production from GECs and subsequent neutrophil infiltration into gastric mucosa by targeting PAR1 lysosomal degradation, leading to gastric inflammation during *H*. *pylori* infection. In the future, it will be interesting to address whether targeting the ARRDC3-PAR1–associated inflammatory cellular networks and/or molecular pathways described here in the context of *H*. *pylori* infection could improve the outcome of the infection, which may lead to the application of novel pharmacological approaches to *H*. *pylori*–associated gastritis.

## Methods

### Patients and specimens.

The gastric biopsy specimens and blood were collected from 70 *H*. *pylori*–infected and 27 uninfected patients who underwent upper esophagogastroduodenoscopy for dyspeptic symptoms at XinQiao Hospital (Supplemental Table 1). *H*. *pylori* infection was determined by [^14^C] urea breath test and rapid urease test of biopsy specimens taken from the antrum, and it was subsequently conformed by real-time PCR for 16s rDNA and serology test for specific anti–*H*. *pylori* Abs. For isolation of human primary GECs, fresh nontumor gastric tissues (at least a 5-cm distance from the tumor site) were obtained from gastric cancer patients who underwent surgical resection and were determined as *H*. *pylori*–negative individuals, as above, at the Southwest Hospital. None of these patients had received chemotherapy or radiotherapy before sampling. Individuals with atrophic gastritis, hypochlorhydria, antibiotics treatment, autoimmune disease, infectious diseases, and multiprimary cancer were excluded.

Abs and other reagents are listed in Supplemental Table 2.

### Mice.

C57BL/6 WT (WT) mice were purchased from the Experimental Animal Center of Third Military Medicine University. C57BL/6 *Arrdc3^–/–^* mice and C57BL/6 *Par1^–/–^* mice were obtained from the Jackson Laboratory. All mice used in experiments were female, except male mice for chimeric experiments, and were viral Ab free for pathogenic murine viruses, were negative for pathogenic bacteria including *Helicobacter* spp. and parasites, and were maintained under specific pathogen–free conditions in a barrier-sustained facility, provided with sterile food (Maintenance Feed) and water.

### Bacteria culture and infection of mice with bacteria.

*H*. *pylori* NCTC 11637 (*cagA*-positive) (WT *H*. *pylori*), *cagA*-KO mutant *H*. *pylori* NCTC 11637 (*ΔcagA*), and *H*. *pylori* 26695 were grown in brain-heart infusion plates containing 10% rabbit blood at 37°C under microaerophilic conditions. For infecting mouse, bacteria were propagated in Brucella broth with 5% FBS with gentle shaking at 37°C under microaerobic conditions. After culture for 1 day, live bacteria were collected and adjusted to 1 × 10^9^ CFU/mL. The mice were fasted overnight and orogastrically inoculated twice at a 1-day interval with 3 × 10^8^ CFU bacteria. *H*. *pylori* infection status and *H*. *pylori*–induced gastritis in murine experiments were confirmed using real-time PCR of *H*. *pylori* 16s rDNA, urease biopsy assays, Warthin-Starry staining, and immunohistochemical staining for *H*. *pylori*, along with evaluation of inflammation by H&E staining (data not shown).

### Generation of BM chimera mice.

The following BM chimeric mice were created: male WT BM→female WT mice, male WT BM→female *Arrdc3^–/–^* mice, male *Arrdc3^–/–^* BM→female WT mice, and male *Arrdc3^–/–^* BM→female *Arrdc3^–/–^* mice. BM cells were collected from the femurs and tibia of donor mice by aspiration and flushing, and they were suspended in PBS at the concentration of 5 × 10^7^/mL. The BM in recipient mice was ablated with lethal irradiation (8 Gy). Then, the animals received i.v. 1.5 × 10^7^ BM cells from donor mice in a volume of 300 μL sterile PBS under the anesthesia. Thereafter, the transplanted BM was allowed to regenerate for 8 weeks before subsequent experimental procedures. To verify successful engraftment and reconstitution of the BM in the host mice, genomic DNA was isolated from tail tissues of each chimera mouse 8 weeks after BM transplantation. Quantitative PCR was performed to detect the *Sry* gene present in the Y chromosome (primers seen in Supplemental Table 3) and mouse β2-microglobulin gene as an internal control. The chimeric rates were calculated on the assumption that the ratio of the Sry/β2-microglobulin gene was 100% in male mice. We confirmed that the chimeric rates were consistently higher than 90%. After BM reconstitution was confirmed, mice were infected with bacteria as described above.

### Chemokine/Ab administration.

One day after infection with WT *H*. *pylori* as described above, WT mice were injected i.p. with 25 μg of recombinant mouse CXCL2, anti-mouse CXCL2 and/or anti-mouse CXCR2 Abs, their isotype control Abs (rat IgG2a and/or IgG2b) (100 μg), anti-Ly6G Abs, or its isotype control Abs (rat IgG2a) (250 μg); this was repeated every week until the mice were sacrificed.

### Evaluation of inflammation.

Mice were sacrificed at the indicated times. The greater curvature of the stomach was cut to perform H&E staining. The intensity of inflammation was evaluated independently by 2 pathologists according to previously established criteria (15, 36).

### Isolation of single cells from tissues.

Fresh tissues were washed 3 times with Hanks’ solution containing 1% FBS, cut into small pieces, collected in RPMI 1640 containing 1 mg/mL collagenase IV and 10 mg/mL DNase I, and then mechanically dissociated by using the gentle MACS Dissociator (Miltenyi Biotec). Dissociated cells were further incubated for 0.5–1 hours at 37°C under continuous rotation. The cell suspensions were then filtered through a 70-μm cell strainer (BD Labware).

### Cell/tissue culture and stimulation.

Primary GECs were purified from gastric tissue single-cell suspensions from uninfected donors or mice with a MACS column purification system using anti-human or -mouse CD326 magnetic beads (Miltenyi Biotec). The sorted primary GECs were cultured in suspension and were used only when their viability was determined to be > 90%. The cells were cultured in complete RPMI 1640 medium supplemented with 10% FBS in a humidified environment containing 5% CO_2_ at 37 °C. Human GEC lines (AGS cells, GES-1 cells, HGC-27 cells) were obtained from American Type Culture Collection (ATCC). Human GEC lines, primary GECs, or primary gastric mucosa tissues from uninfected donors or mice were infected with WT *H*. *pylori* or *ΔcagA* at a multiplicity of infection (MOI) of 100 for 24 hours. AGS cells were also infected with WT *H*. *pylori* at different MOI (24 hours) or at the indicated time points (MOI = 100). For signal pathway inhibition experiments, AGS cells were pretreated with 20 μM of U0126, Wortmannin, AG490, SB203580, or SP600125 for 2 hours. For ARRDC3/PAR1/SP1 inhibition experiments, AGS cells were pretreated with *ARRDC3/PAR1/SP1* siRNA, NC (40 nM), or lipofectamine 3000 only (mock) for 24 hours. In some cases, AGS cells were transfected with plasmids pcDNA3.1 or *cagA*-pcDNA3.1 by using lipofectamine 3000 according to the manufacturer’s protocols. At 24 hours after transfection, cells were treated with or without U0126 or Wortmannin (20 μM) or DMSO control for 2 hours and cultured for an additional 24 hours. In some cases, AGS cells were pretreated with or without Bafilomycin A1 (1 μM) for 30 minutes, and further incubated with WT *H*. *pylori* (MOI = 100) for 24 hours in the presence or absence of Bafilomycin A1. For transwell assays, AGS cells were added to the lower chamber, and WT *H*. *pylori* or *H*. *pylori* 26695 (MOI = 100) were placed into the lower or the upper chambers of transwells (0.4-μm pore) and then incubated for 24 hours. After coculture, cells were collected for real-time PCR, Western blot, immunofluorescence, and co-IP, and the culture supernatants were harvested for ELISA.

### Luciferase reporter assay.

Promoter constructs containing the region from –2000 to 0 of the *ARRDC3* gene were amplified from human genomic DNA by PCR. The amplified full length or fragments were cloned into the NheI and HindIII sites of the pGL3-basic vector, respectively, by Sangon Biotech. Mutant of –100/0 sequences were synthesized and cloned into the NheI and HindIII sites of the pGL3-basic vector. The mutant sequence was 5′-GGGCAAGGGAGCGAGCGCGGCGCGGCGCGGCGCGGGAGGGGGCGCGCAGGG GCAGCCGCGGCCTGCGCCTGCGCACTGGGGTTGTTTTTC-3′ (deleted SP1 binding site, 5′-AGGGCGGACA-3′). For the luciferase reporter assay, cells were seeded in 24-well plates and were transfected when reaching approximately 80% confluence with the constructed luciferase reporter vector for 4 hours. Lipofectamine 2000 was used to transfect AGS cells according to the manufacturer’s protocols. Luciferase activity was measured to assess promoter activity after WT *H*. *pylori* (cells pretreated with or without U0126 or Wortmannin before WT *H*. *pylori* infection) or Δ*cagA* infection (MOI = 100) for 24 hours by the Dual-Luciferase Reporter assay following the manufacturer’s protocol. Luciferase activity was normalized to Renilla luciferase activity.

### Co-IP assay.

IP assay was performed using a Pierce Classic Magnetic IP/Co-IP Kit following the manufacturer’s protocol. Whole-cell extracts were lysed in IP Lysis/Wash Buffer in the presence of protease inhibitor. After centrifugation for 10 minutes at 13,000 *g* at 4°C, supernatants were collected and incubated with rabbit anti-ARRDC3 mAb or rabbit IgG at 4°C overnight. After overnight incubation, the protein A/G magnetic beads (washed 3 times with IP Lysis/Wash Buffer) were incubated with total cell extracts with gentle shaking for 1 hour at room temperature. Then, the beads were washed 3 times with IP Lysis/Wash Buffer and resuspended in 50 μL of 1% (w/v) SDS sample buffer and boiled at 97°C for 10 minutes. The proteins were separated by SDS-PAGE (10% SDS) and transferred to a PVDF membrane for immunoblot detection with mouse anti-PAR1 mAb.

### Chemotaxis assay.

Human CD45^+^CD11b^+^CD14^–^CD66b^+^ neutrophils or mouse CD45^+^CD11b^+^Ly6C^–^Ly6G^+^ neutrophils from blood of *H*. *pylori*–infected donors or WT *H*. *pylori*–infected mice (day 28 p.i.) were sorted by FACS (FACSAria II; BD Biosciences). AGS cells were pretreated with *ARRDC3/PAR1* siRNA or NC (both at 40 nM) for 24 hours, and then stimulated with WT *H*. *pylori* or *ΔcagA* (MOI = 100) for 24 hours. The culture supernatants were collected and used as a source of chemoattractants in a human neutrophil chemotaxis assay. In another set of experiments, mouse primary GECs were purified from gastric tissue single-cell suspensions of uninfected mice with a MACS column purification system using anti–mouse CD326 magnetic beads (Miltenyi Biotec), and then stimulated with WT *H*. *pylori* or *ΔcagA* (MOI = 100) for 24 hours. The culture supernatants were collected as mentioned above. These culture supernatants were then used as source of as chemoattractants in a mouse neutrophil chemotaxis assay.

In a chemotaxis assay, sorted neutrophils (1 × 10^5^) were transferred into the upper chambers of transwells (5-μm pore). CXCL2 (100 ng/mL) and culture supernatants from various cultures were placed in the lower chambers. After 6 hours of culture, migration was quantified by counting cells in the lower chamber and cells adhering to the bottom of the membrane. In some cases, blocking Abs for human/mouse CXCL2 (20 μg/mL) or corresponding control IgG1/IgG2b (20 μg/mL) were added into culture supernatants, and blocking Abs for human/mouse CXCR2 (20 μg/mL) or corresponding control IgG2a (20 μg/mL) were added into neutrophil suspensions and incubated for 2 hours before chemotaxis assay.

### IHC.

Paraformaldehyde-fixed and paraffin-embedded samples were cut into 5-μm sections. For immunohistochemical staining, the sections were incubated with rabbit anti–human or –mouse ARRDC3, followed by HRP-conjugated anti–rabbit IgG and later its substrate diaminobenzidine. All the sections were finally counterstained with hematoxylin and examined using a microscope (Nikon Eclipse 80i).

### Immunofluorescence.

Paraformaldehyde-fixed cryostat tissue sections or AGS cells were washed in PBS, blocked for 30 minutes with 20% goat serum in PBS, and stained for ARRDC3, ARRDC3 and CD326, ARRDC3 and H^+^/K^+^ ATPase, ARRDC3 and pepsinogen II, or ARRDC3 and PAR1 (together with LysoTracker Deep Red). Slides were examined with a confocal fluorescence microscope (LSM 510 META, Zeiss).

### Real-time PCR.

DNA of the biopsy specimens were extracted with QIAamp DNA Mini Kit and RNA of biopsy specimens, and cultured cells were extracted with TRIzol reagent. The RNA samples were reversed transcribed into cDNA with PrimeScript RT reagent Kit. Real-time PCR was performed on an IQ5 (Bio-Rad) with Real-time PCR Master Mix according to the manufacturer’s specifications. The mRNA expression of 16s rDNA, *cagA*, *ARRDC3/Arrdc3*, *Il6*, *Tnfa*, chemokine, arrestin, and *SP1* genes was measured using the TaqMan and/or SYBR green method with the relevant primers (Supplemental Table 3). For mice, mouse β2-microglobulin mRNA level served as a normalizer, and its level in the stomach of uninfected or WT mice served as a calibrator. For humans, human β-actin mRNA level served as a normalizer, and its level in the unstimulated cells or stomach of uninfected donors served as a calibrator. The relative gene expression was expressed as fold change of relevant mRNA calculated by the ΔΔCt method.

### Flow cytometry.

Cell surface markers were stained with specific or isotype control Abs and analyzed by multicolor flow cytometry on a FACSCanto II (BD Biosciences). Data were analyzed with FlowJo (Tree Star Inc.) or FACSDiva software (BD Biosciences).

### ELISA.

Isolated human and mouse gastric tissues were homogenized in 1 mL sterile Protein Extraction Reagent and centrifuged (13,000 g, 4°C, 10 minutes). Tissue supernatants were collected for ELISA. Concentrations of CXCL2, IL-6, and TNF-α in the tissue supernatants and concentrations of CXCL2 in the GEC culture supernatants were determined using ELISA kits according to the manufacturer’s instructions.

### Western blot analysis.

Western blots were performed on 10%–15% SDS-PAGE gel–transferred PDF membranes with equivalent amounts of cell or tissue lysate proteins for each sample. Skim milk (5%) was used for blocking the PDF membranes. Mouse ARRDC3 and PAR1 was detected with rabbit anti-ARRDC3 Abs and rabbit anti-PAR1 Abs; human ARRDC3, PAR1, ERK1/2, p-ERK1/2, AKT, and p-AKT were detected with rabbit anti-ARRDC3 Abs, rabbit anti-PAR1 Abs, rabbit anti-ERK1/2 Abs, rabbit anti–p-ERK1/2 Abs, rabbit anti-AKT Abs, and rabbit anti–p-AKT Abs, respectively. This was followed by incubation with HRP-conjugated secondary Abs. Bound proteins were visualized by using SuperSignal West Dura Extended Duration Substrate kit.

### Statistics.

Results are expressed as mean ± SEM. Two-tailed Student *t* test was generally used to analyze the differences between 2 groups, but when the variances differed, the Mann-Whitney *U* test was used. Inflammation score data were analyzed by the Mann-Whitney *U* test. For multiple comparisons, 1-way ANOVA was used. Correlations between parameters were assessed using Pearson correlation analysis and linear regression analysis, as appropriate. SPSS statistical software (version 13.0) was used for all statistical analyses. All data were analyzed using 2-tailed tests, and *P* < 0.05 was considered statistically significant. Raw data from each array were analyzed using TwoClassDif.

### Study approval.

All breeding and experiments were undertaken with review and approval from the Animal Ethical and Experimental Committee of Third Military Medical University. The experiments involving human blood and stomach biopsy samples were approved by the Ethics Committee of XinQiao Hospital and Southwest Hospital of Third Military Medical University. The written informed consent was obtained from each subject.

## Author contributions

All listed authors participated meaningfully in the study, and they have seen and approved the submission of this manuscript. YZ designed the research. YGL and YST participated in performing the research, analyzing the data, and initiating the original draft of the article. YZ and WC revised the manuscript. ZGS, PC, CJH, YPL, and FYM participated in performing the research and collecting the data. QMZ, NY, SMY, and YLZ contributed reagents and human clinical samples. YZ, QMZ, and NY supervised the studies, analyzed the data, and wrote the manuscript.

## Supplementary Material

Supplemental data

## Version 1 07/07/2020

In-Press Preview

## Version 2 08/06/2020

Electronic publication
