# Supplementary material for: Arrestin domain containing 3 promotes Helicobacter pylori–associated gastritis by regulating protease-activated receptor 1
Source: JCI Insight. 2020 Aug 6;5(15):e135849. doi: 10.1172/jci.insight.135849 (PMC7455081; doi:10.1172/jci.insight.135849)
Supplement: Supplemental data [file jciinsight-5-135849-s100.pdf]

Supplemental Figure 1

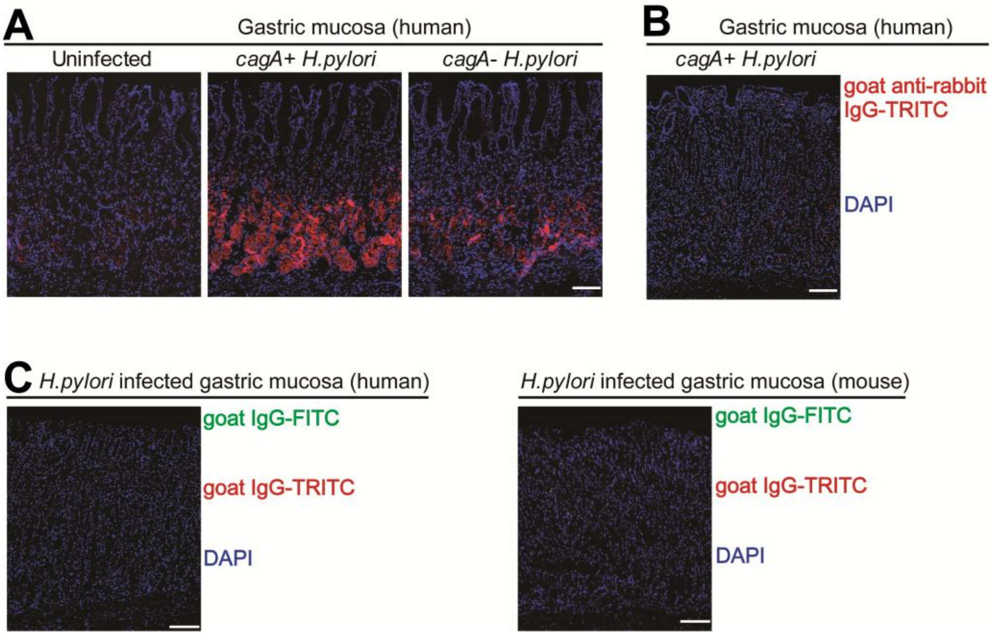

ARRDC3 is increased in gastric mucosa of *H. pylori*-infected patients and mice. (A) ARRDC3 expression in gastric mucosa of *cagA*$^+$ *H. pylori*-infected, *cagA*$^-$ *H. pylori*-infected, and uninfected donors was analyzed by immunofluorescence staining. Scale bars: 100 microns. (B and C) Representative immunofluorescence staining images showing only secondary antibody staining controls in gastric mucosa of *H. pylori*-infected patients or *H. pylori*-infected mice. Scale bars: 100 microns.

Supplemental Figure 2

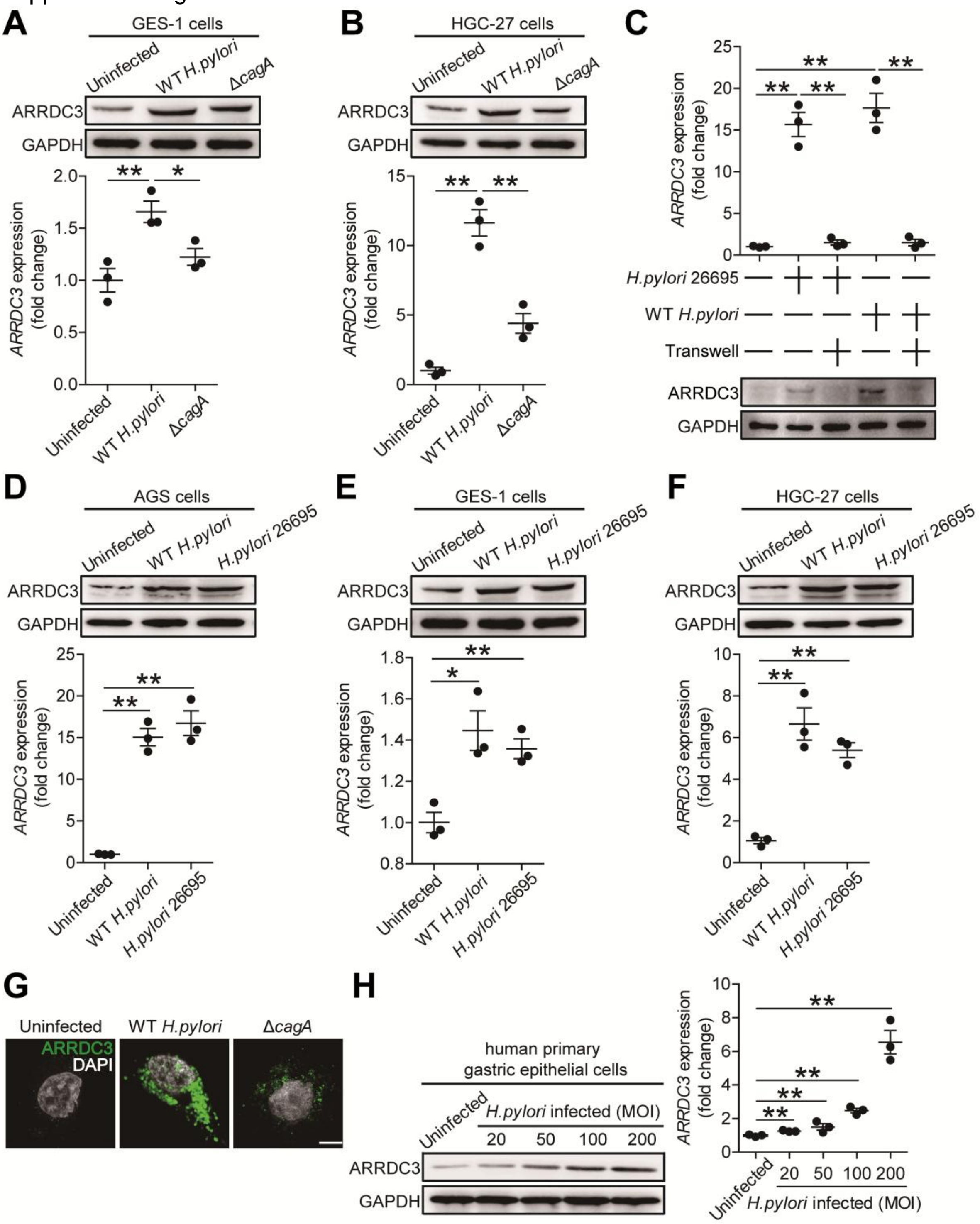

H. pylori stimulates gastric epithelial cells express ARRDC3. (A and B) *ARRDC3* expression and ARRDC3 protein in WT *H. pylori*-infected, *ΔcagA*-infected, and uninfected GES-1 cells (A) and HGC-27 cells (B) (MOI=100, 24 h) was analyzed by real-time PCR and western blot (n=3). (C) *ARRDC3* expression and ARRDC3 protein in AGS cells infected with WT *H. pylori* or *H. pylori* 26695 (MOI=100, 24 h) was assessed by transwell assay and analyzed by real-time PCR and western blot (n=3) as described in Materials and Methods. (D-F) *ARRDC3* expression and ARRDC3 protein in WT *H. pylori*-infected, *H. pylori* 26695-infected, and uninfected AGS cells (D), GES-1 cells (E) and HGC-27 cells (F) (MOI=100, 24 h) was analyzed by real-time PCR and western blot (n=3). (G) Representative immunofluorescence staining images showing ARRDC3 expression (green) in uninfected, WT *H. pylori*-infected and *ΔcagA*-infected AGS cells (MOI=100, 6 h). Scale bars: 1 micron. (H) *ARRDC3* expression and ARRDC3 protein in WT *H. pylori*-infected and uninfected human primary gastric epithelial cells with different MOI (24 h) were analyzed by real-time PCR and western blot (n=3). Data are representative of 2 independent experiments. Data are mean ± SEM and analyzed by 1-way ANOVA. Western blot results are run in parallel and contemporaneously. *P<0.05, **P<0.01 for groups connected by horizontal lines.

Supplemental Figure 3

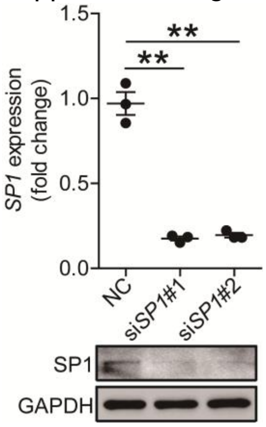

SP1 siRNA (si*SP1*#1, si*SP1*#2) or non-specific control siRNA (NC) pre-treated AGS cells were infected with WT *H. pylori* (MOI=100) for 24 h. *SP1* expression and SP1 protein were analyzed by real-time PCR and western blot (n=3). Data are representative of 2 independent experiments. Data are mean ± SEM and analyzed by 1-way ANOVA. Western blot results are run in parallel and contemporaneously. **P*<0.05, ***P*<0.01 for groups connected by horizontal lines.

Supplemental Figure 4

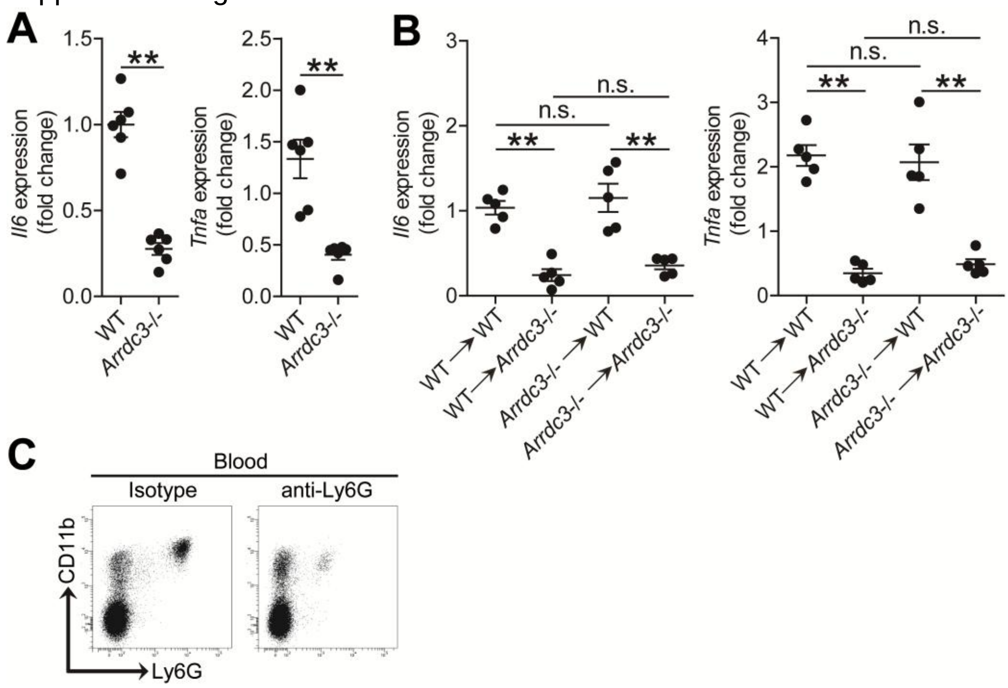

ARRDC3 promotes *Il6* and *Tnfa* expression during *H. pylori* infection. (A and B) *Il6* and *Tnfa* expression in gastric mucosa of WT *H. pylori*-infected WT and *Arrdc3*$^{-/-}$ mice (n=6) (A), or in gastric mucosa of WT *H. pylori*-infected BM chimera mice (n=5) (B) on day 28 p.i. was compared. (C) Neutrophil depletion was confirmed by flow cytometric analysis of whole blood. Data are representative of 2 independent experiments. Data are mean ± SEM and analyzed by Student *t* test, Mann-Whitney U test and 1-way ANOVA. **P*<0.05, ***P*<0.01, n.s. *P*>0.05 for groups connected by horizontal lines.

Supplemental Figure 5

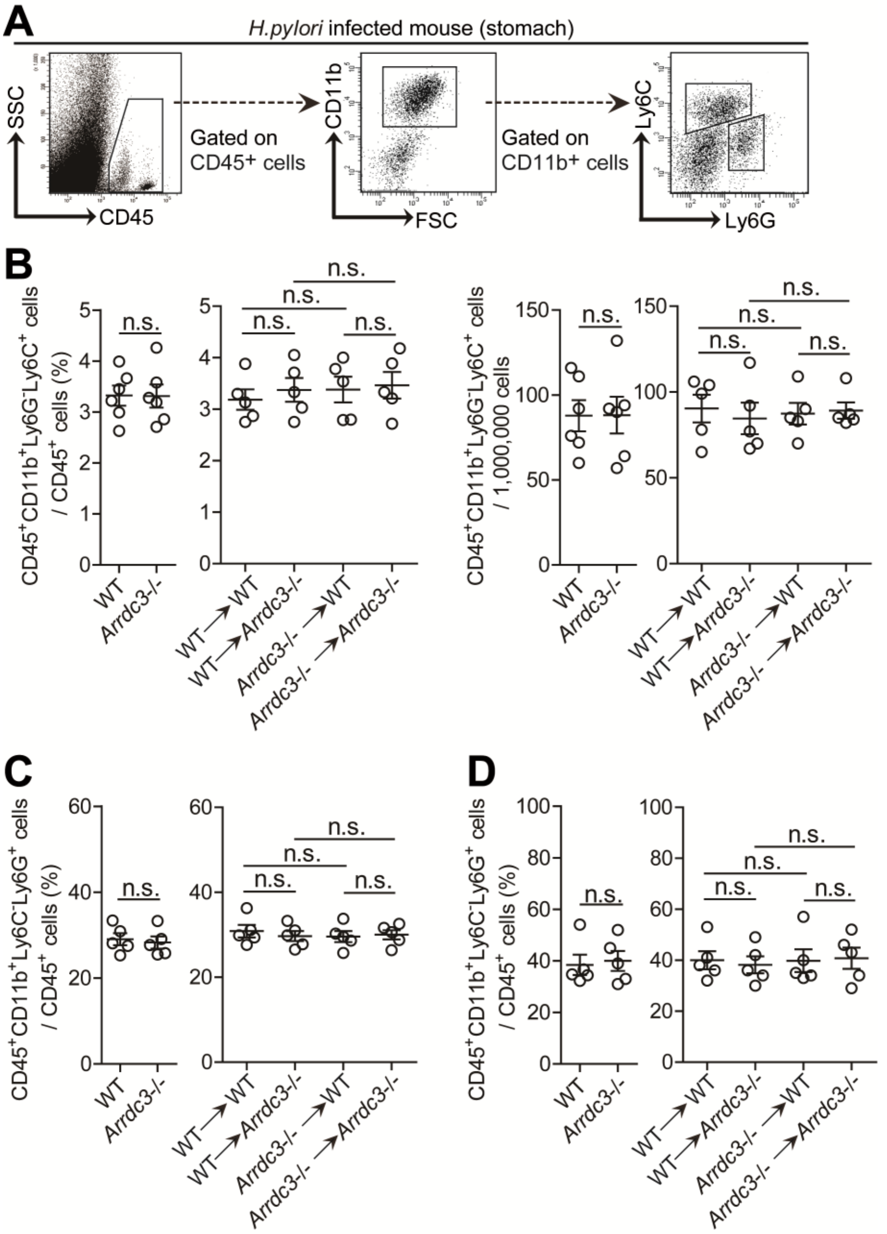

ARRDC3 has no effect on monocyte accumulation during *H. pylori* infection. (A) Dot plots of CD45$^+$CD11b$^+$Ly6C$^-$Ly6G$^+$ neutrophils and CD45$^+$CD11b$^+$Ly6G$^-$Ly6C$^+$ monocytes by gating on CD45$^+$ cells and following CD11b$^+$ cells in gastric mucosa of WT *H. pylori*-infected mice on day 28 p.i.. (B) CD45$^+$CD11b$^+$Ly6G$^-$Ly6C$^+$ monocyte level in gastric mucosa of WT *H. pylori*-infected WT and *Arrdc3*$^{-/-}$ mice (n=6), or in gastric mucosa of WT *H. pylori*-infected BM chimera mice (n=5) on day 28 p.i. was compared. (C and D) CD45$^+$CD11b$^+$Ly6C$^-$Ly6G$^+$ neutrophil level in blood (C) or bone marrow (D) of WT *H. pylori*-infected WT and *Arrdc3*$^{-/-}$ mice or of WT *H. pylori*-infected BM chimera mice on day 28 p.i. was compared (n=5). Data are representative of 2 independent experiments. Data are mean ± SEM and analyzed by Student *t* test, Mann-Whitney U test and 1-way ANOVA. n.s. P>0.05 for groups connected by horizontal lines.

Supplemental Figure 6

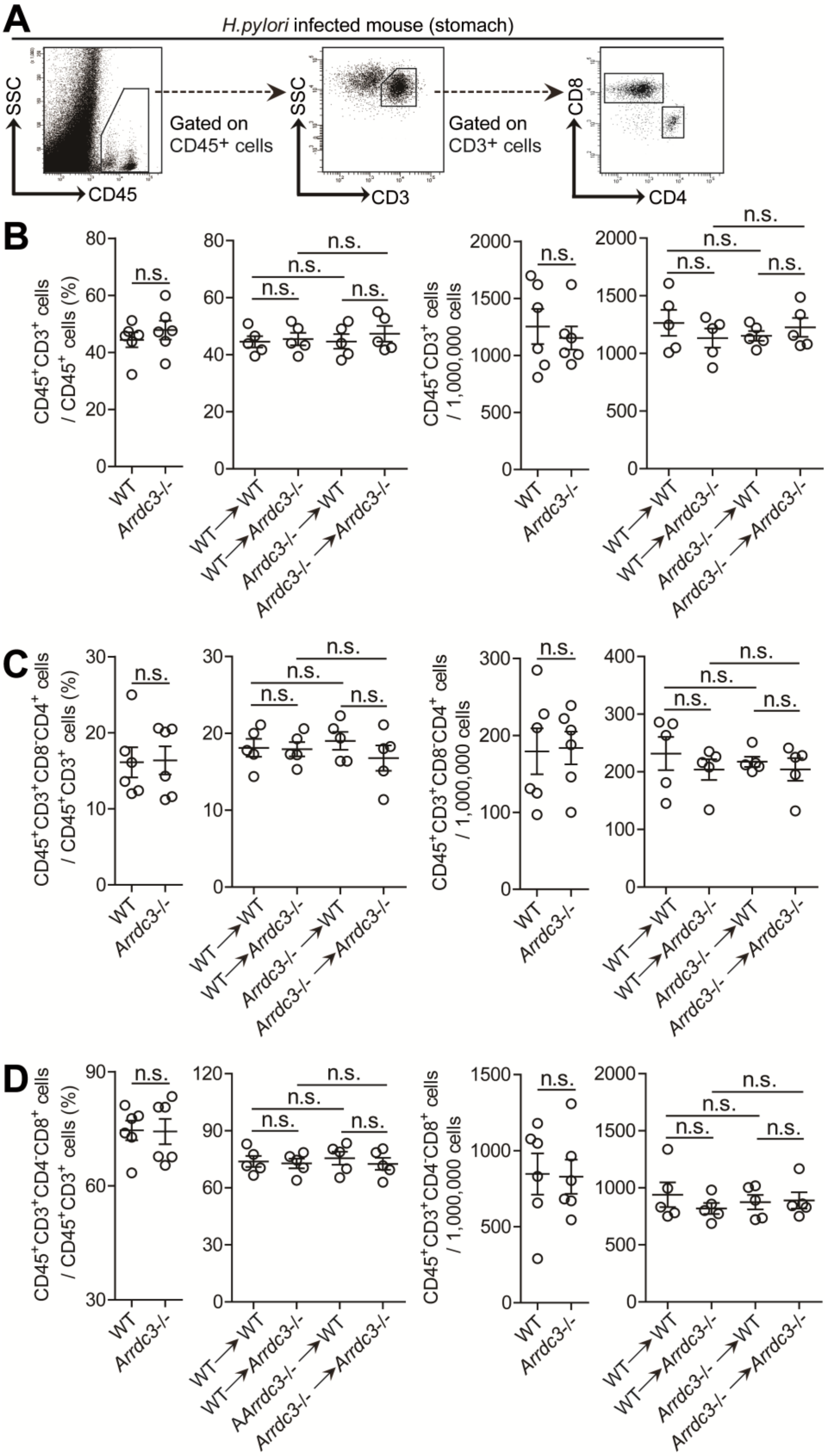

ARRDC3 has no effect on T cell accumulation during *H. pylori* infection. (A) Dot plots of $CD45^+CD3^+$ T cells, $CD45^+CD3^+CD8^-CD4^+$ T cells and $CD45^+CD3^+CD4^-CD8^+$ T cells by gating on $CD45^+$ cells and following $CD3^+$ cells in gastric mucosa of WT *H. pylori*-infected mice on day 28 p.i.. (B-D) $CD45^+CD3^+$ T cell level (B), $CD45^+CD3^+CD8^-CD4^+$ T cell level (C) and $CD45^+CD3^+CD4^-CD8^+$ T cell level (D) in gastric mucosa of WT *H. pylori*-infected WT and *Arrdc3*$^{-/-}$ mice (n=6), or in gastric mucosa of WT *H. pylori*-infected BM chimera mice (n=5) on day 28 p.i. was compared. Data are representative of 2 independent experiments. Data are mean ± SEM and analyzed by Student *t* test, Mann-Whitney U test and 1-way ANOVA. n.s. *P*>0.05 for groups connected by horizontal lines.

Supplemental Figure 7

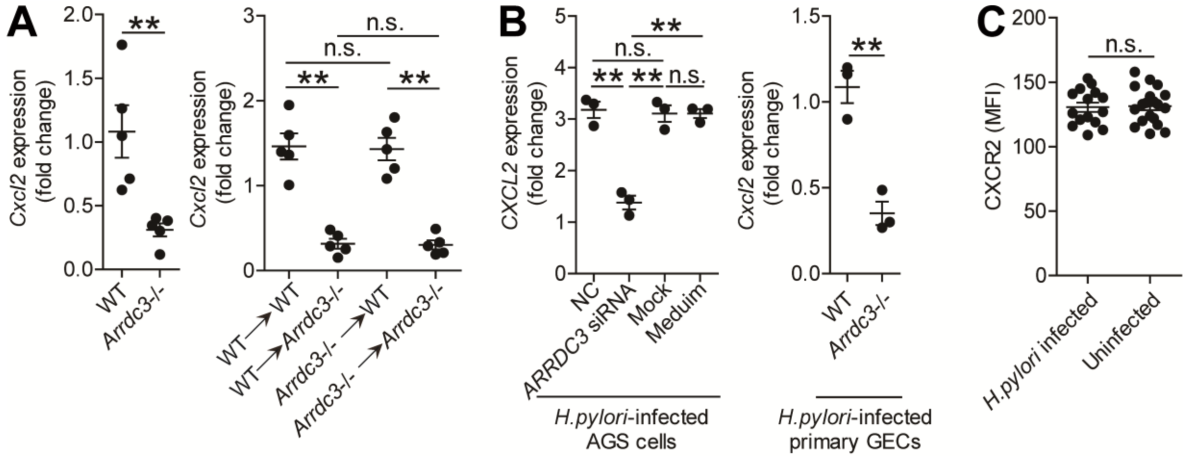

ARRDC3 promotes *CXCL2*/*Cxcl2* expression in vivo and in vitro during *H. pylori* infection. (A) *Cxcl2* expression in gastric mucosa of WT *H. pylori*-infected WT and *Arrdc3*$^{-/-}$ mice, or in gastric mucosa of WT *H. pylori*-infected BM chimera mice (n=5) on day 28 p.i. was compared. (B) *ARRDC3* siRNA, non-specific control siRNA (NC), or lipo3000 only (Mock) pre-treated AGS cells or AGS cells without treatment (medium), and primary gastric epithelial cells (GECs) from uninfected WT and *Arrdc3*$^{-/-}$ mice were infected with WT *H. pylori* (MOI=100) for 24 h. *CXCL2*/*Cxcl2* expression was measured in cells by real-time PCR (n=3). (C) CXCR2 expression on human CD45^{+}CD11b^{+}CD14^{-}CD66b^{+} neutrophils in blood of *H. pylori*-infected patients (n=15) or uninfected donors (n=19) was compared. Data are representative of 2 independent experiments. Data are mean ± SEM and analyzed by Student *t* test, Mann-Whitney U test and 1-way ANOVA. ***P*<0.01, n.s. *P*> 0.05 for groups connected by horizontal lines. MFI, Median Fluorescence Intensity.

Supplemental Table 1.Clinical characteristics of patients

Variables	*H. pylori*-infected	Uninfected
Age (median, range)	(41 year, 22–76 years)	(39 year, 24–65 years)
Sex (male/female)	49/21	15/12

Exclusion criteria were: previous treatment for *H. pylori* infection, use of inhibitors of acid secretion and/or antibiotics during the 2 months before the study, use ofanticoagulant drugs in the last week, gastrointestinal malignancy, severe concomitant cardiovascular, respiratory or endocrine diseases, clinically significant renal or hepatic disease, haematological disorders, previous gastro-oesophageal surgery, history of allergy to any of the drug used in the study, pregnancy or lactation, alcohol abuse, drug addiction, severe neurological or psychiatric disorders, and long-term use of corticosteroids or anti-inflammatory drugs.

Supplemental Table 2.Antibodies and other reagents

Antibodies and reagents	Manufacturers
Antibodies for flow cytometry	
anti-mouse CD45-PE-Cy7(stock no. 103113)	Biolegend
anti-mouse CD11b-PerCP-Cy5.5(stock no. 101227)	Biolegend
anti-mouse Ly6G-FITC(stock no. 127605)	Biolegend
anti-mouse Ly6C-PE(stock no. 128007)	Biolegend
anti-mouse CD3-APC(stock no. 100235)	Biolegend
anti-mouse CD8-PerCP-Cy5.5(stock no. 100733)	Biolegend
anti-mouse CD4-PE(stock no. 100407)	Biolegend
anti-mouse CXCR2-APC(stock no. 149311)	Biolegend
anti-human CD45-PE-Cy7(stock no. 368531)	Biolegend
anti-human CD11b-PerCP-Cy5.5(stock no. 101227)	Biolegend
anti-human CD14-PE(stock no. 367103)	Biolegend
anti-human CD66b-FITC(stock no. 305103)	Biolegend
anti-human CXCR2-APC(stock no. 320710)	Biolegend
Antibodies for immunohistochemical staining	
rabbit anti-human/mouse ARRDC3(stock no. ab64817)	Abcam
horseradish peroxidase anti-rabbit IgG(stock no. ZDR-5307)	Zhongshan Biotechnology
Antibodies for immunofluorescence	
rabbit anti-human/mouse ARRDC3(stock no. ab64817)	Abcam
mouse anti-human/mouse CD326 (EpCAM)(stock no. 323/A3)	Invitrogen
mouse anti-human/mouse H^+/K^+ ATPase(stock no. ab2866)	Abcam
rabbit anti-human/mouse pepsinogen II(stock no. ab180709)	Abcam
mouse anti-human/mouse PAR1(stock no. 611522)	BD
mouse anti-human PAR1(stock no. ab233741)	Abcam
LysoTracker™ Deep Red	Invitrogen
goat anti-rabbit-TRITC(stock no. ZF-0316)	Zhongshan Biotechnology
goat anti-rabbit-FITC(stock no. ZF-0311)	Zhongshan Biotechnology
goat anti-mouse-TRITC(stock no. ZF-0313)	Zhongshan Biotechnology
goat anti-mouse-FITC(stock no. ZF-0312)	Zhongshan Biotechnology
Antibodies for neutralizing and blocking	
anti-human CXCL2 (Mouse IgG1)(stock no. ab89324)	Abcam
Mouse IgG1 Isotype Control(stock no. ab81216)	Abcam
anti-human CXCR2 (Mouse IgG1)(stock no. MAB331)	R&D Systems
Mouse IgG2a Isotype Control(stock no. MAB003)	R&D Systems
anti-mouse CXCL2 (Rat IgG2b)(stock no. MAB452)	R&D Systems

Rat IgG2b Isotype Control(stock no. MAB0061)	R&D Systems
anti-mouse CXCR2 (Rat IgG2a)(stock no. MAB2164)	R&D Systems
Rat IgG2a Isotype Control(stock no. MAB006)	R&D Systems
anti-mouse Ly6G (Rat IgG2a)(stock no. 127632)	Biolegend
Rat IgG2a Isotype Control(stock no. 402301)	Biolegend
Antibodies for western blot	
rabbit anti-human/mouseARRDC3(stock no. ab64817)	Abcam
rabbit anti-human ERK1/2(stock no. 4695)	Cell signaling technology
rabbit anti-human p-ERK1/2(stock no. 4370)	Cell signaling technology
rabbit anti-human AKT(stock no. 4685)	Cell signaling technology
rabbit anti-human p-AKT(stock no. 4060)	Cell signaling technology
rabbit anti-human AKT(stock no. ab214167)	Abcam
rabbit anti-human p-AKT(stock no. ab251150)	Abcam
rabbit anti-human JNK(stock no. ab225572)	Abcam
rabbit anti-human p-JNK(stock no. ab239886)	Abcam
rabbit anti-human/mouse PAR1(stock no. ab32611)	Abcam
rabbit anti-human/mouse GAPDH(stock no. RM2002)	Beijing Ray Antibody Biotech
rabbit anti-human/mouse GAPDH(stock no. ab199554)	Abcam
rabbit anti-human/mouse GAPDH(stock no. ab186930)	Abcam
Antibodies for co-inmunoprecipitation	
rabbit anti-human ARRDC3(stock no. ab64817)	Abcam
rabbit IgG(stock no. ab172730)	Abcam
mouse anti-human PAR1(stock no. ab233741)	Abcam
ELISA kits	
human CXCL2	Abcam
mouse CXCL2	R&D Systems
mouse IL-6	Abcam
mouse TNF-α	Abcam
Reagents for signaling pathways inhibition	
MEK-1 and MEK-2 inhibitorU0126	Merck Millipore
IκBαinhibitor BAY 11-7082	Merck Millipore
JNK inhibitor SP600125	Merck Millipore
MAPK inhibitor SB202190	Merck Millipore
PI3K-AKT inhibitor Wortmannin	Merck Millipore
Human CD326 microbeads	MiltenyiBiotec
Mouse CD326 microbeads	MiltenyiBiotec
5-μm pore size Transwells	Corning

0.4-μm pore size Transwells	Corning
Collagenase IV	Gibco
DNase I	Sigma-Aldrich
DMSO	Sigma-Aldrich
Bafilomycin A1	Sigma-Aldrich
Protein Extraction Reagent	Pierce
SuperSignal® West Dura Extended Duration Substrate kit	Thermo
Fetal bovine serum (FBS)	Invitrogen
Penicillin/Streptomycin	Gibco
RPMI-1640	Hyclone
DMEM/F12 (1:1)	Hyclone
Ficoll-Paque Plus	GE Healthcare
lyses solution	TIANGEN
TRIzol reagent	Invitrogen
Lipofectamine™ 3000 Transfection Reagent	Invitrogen
Lipofectamine™ 2000 Transfection Reagent	Invitrogen
Lipofectamine™ RNAiMAX Transfection Reagent	Invitrogen
QIAamp DNA Mini Kit	QIAGEN
PrimeScriptTM RT reagent Kit	TaKaRa
Real-time PCR Master Mix	Toyobo
pGL3-basic vector	Promega
Dual-Luciferase Reporter assay Kit	Promega
Pierce™ Classic Magnetic IP/Co-IP Kit	R&D Systems
Protease inhibitor	Thermo Scientific
Recombinant human CXCL2	Roche
Recombinant mouse CXCL2	R&D Systems

APC-Cy7, allophycocyanin-cyanin 7; PE-Cy7, phycoerythrin-cyanin 7; FITC, Fluorescein isothiocyanate; PE, phycoerythrin; PerCP-Cy5.5, peridinchlorophyl protein-cyanin 5.5; APC, allophycocyanin; IL, interleukin.

SupplementalTable 3.Primer and probe sequences for real-time PCR analysis

Gene	Primer or probe	Sequence 5′→3′
H. pylori 16s rDNA	forward	TTTGTTAGAGAAGATAATGACGGTATCTAAC
	reverse	CATAGGATTTCACACCTGACTGACTATC
	probe	CGTGCCAGCAGCCGCGGT
Mouse *β2-microglobulin*	forward	CCTGCAGAGTTAAGCATGCCAG
	reverse	TGCTTGATCACATGTCTCGATCC
	probe	TGGCCGAGCCCAAGACCGTCTAC
H. pyloricagA	forward	GAGTCATAATGGCATAGAACCTGAA
	reverse	TTGTGCAAGAAATTCCATGAAA
Mouse *Sry*	forward	TGGGACTGGTGACAATTGTC
	reverse	GAGTACAGGTGTGCAGCTCT
Human *GAPDH*	forward	ACCCAGAAGACTGTGGATGG
	reverse	CAGTGAGCTTCCCGTTCAG
Mouse *β-actin*	forward	AGTGTGACGTTGACATCCGT
	reverse	GCAGCTCAGTAACAGTCCGC
Mouse *Arrdc3*	forward	CCGTTTGGTAGCAGAACCTC
	reverse	TCCTCCGTTACAACCTCTGC
Mouse *Ccl1*	forward	ATGGCACTGATGTGCCTGCT
	reverse	GGTGGAGGACTGAGGGAAA
Mouse *Ccl2*	forward	TCACCTGCTGCTACTCATTCA
	reverse	CACTGTCACACTGGTCACTCC
Mouse *Ccl3*	forward	TTCTCTGTACCATGACACTCTGC
	reverse	CGTGGAATCTTCCGGCTGTAG

Mouse *Ccl4*	forward	TGTCTGCCCTCTCTCTCCTCT
	reverse	AGCAAGGACGCTTCTCAGTGA
Mouse *Ccl5*	forward	GCTGCTTTGCCTACCTCTCC
	reverse	TCGAGTGACAAACACGACTGC
Mouse *Ccl6*	forward	CCAAGACTGCCATTTCATTC
	reverse	AAGCAATGACCTTGTTCCCA
Mouse *Ccl7*	forward	ATGGAAGTCTGCGCTGAAG
	reverse	ACATGAGGTCTCCAGAGCTTT
Mouse *Ccl8*	forward	ACGCTAGCCTTCACTCCAAAA
	reverse	TTCCAGCTTTGGCTGTCTCTT
Mouse *Ccl9*	forward	TGGCATATCTGGCTTTGTCA
	reverse	ATGGCTGTAGCTCAAGATGGT
Mouse *Ccl11*	forward	TCCACAGCGCTTCTATTCCT
	reverse	GCAGTTCTTAGGCTCTGGGTT
Mouse *Ccl12*	forward	TCGAAGTCTTTGACCTCAACA
	reverse	GGGAACTTCAGGGGGAAATA
Mouse *Ccl19*	forward	ACTTGCACTTGGCTCCTGAA
	reverse	AGTCTTCCGCATCATTAGCA
Mouse *Ccl20*	forward	GCAAGCGTCTGCTCTTCCTT
	reverse	TTAGGCTGAGGAGGTTCACA
Mouse *Ccl21*	forward	GATGATGACTCTGAGCCTCCT
	reverse	TTCTGCACCCAGCCTTCCT

Mouse *Ccl22*	forward	TGGCAATTCAGACCTCTGATG
	reverse	TTGCTGGAATGGCAGAAGAA
Mouse *Ccl24*	forward	TCATCTTGCTGCACGTCCTTT
	reverse	TAAACCTCGGTGCTATTGCCA
Mouse *Ccl25*	forward	TCTCAGGACCAGAAAGGCATT
	reverse	TGGCGGAAGTAGAATCTCACA
Mouse *Ccl27*	forward	AGGCTGAGTGAGCATGATGGA
	reverse	TTGGCGTTCTAACCACCGA
Mouse *Ccl28*	forward	GCTGTGTGTGTGGCTTTTCAA
	reverse	TACCTCTGAGGCTCTCATCCA
Mouse *Cx3cl1*	forward	TGGCTTTGCTCATCCGCTATCAG
	reverse	CGTCTGTGCTGTGTCGTCTCC
Mouse *Cxcl1*	forward	ACCCAAACCGAAGTCATAG
	reverse	TTGTATAGTGTTGTCAGAAGC
Mouse *Cxcl2*	forward	ACTTCAAGAACATCCAGAG
	reverse	CTTTCCAGGTCAGTTAGC
Mouse *Cxcl3*	forward	CAGCCACACTCCAGCCTA
	reverse	CACAACAGCCCCTGTAGC
Mouse *Cxcl4*	forward	AGCGATGGAGATCTTAGCTGTGT
	reverse	CCAGGCTGGTGATGTGCTTAA
Mouse *Cxcl5*	forward	AGTCAAGAATCATTGGTTGTTAACCTT
	reverse	TCCGGAGACAATGCAATAGTCA

Mouse *Cxcl7*	forward	GGAGTTCACTGTGCTGATGTGGA
	reverse	CACAGATGAAGCAGCTGGTCAGTAA
Mouse *Cxcl9*	forward	ACAAATCCCTCAAAGACCTCAAACAG
	reverse	ATCTCCGTTCTTCAGTGTAGCAATG
Mouse *Cxcl10*	forward	TGAAAGCGTTTAGCCAAAAAAGG
	reverse	AGGGGAGTGATGGAGAGAGG
Mouse *Cxcl12*	forward	CCTCCAAACGCATGCTTCA
	reverse	ACTCTCCTCCCTTCCATTGCA
Mouse *Cxcl13*	forward	CAGGCCACGGTATTCTGGA
	reverse	CAGGGGGCGTAACTTGAATC
Mouse *Cxcl14*	forward	GCTTCATCAAGTGGTACAAT
	reverse	CTGGCCTGGAGTTTTTCTTTCCAT
Mouse *Cxcl15*	forward	CTAGGCATCTTCGTCCGTCC
	reverse	TTGGGCCAACAGTAGCCTTC
Mouse *Cxcl16*	forward	AAACATTTGCCTCAAGCCAGT
	reverse	GTTTCTCATTTGCCTCAGCCT
Mouse *Cxcl17*	forward	ATGAAGCTTCTAGCCTCTCCC
	reverse	CTATAAGGGCAGCGCAAAGCTTGC
Mouse *Tnfa*	forward	ATGTCTCAGCCTCTTCTCATTC
	reverse	GCTTGTCACTCGAATTTTGAGA
Mouse *Il6*	forward	CTCCCAACAGACCTGTCTATAC
	reverse	CCATTGCACAACTCTTTTCTCA

Human *ARB1*	forward	AAAGGGACCCGAGTGTTCAAG
	reverse	CGTCACATAGACTCTCCGCT
Human *ARB2*	forward	TCCATGCTCCGTCACACTG
	reverse	ACAGAAGGCTCGAATCTCAAAG
Human *ARRDC1*	forward	AGTTCCTGCTTCCTGCCACT
	reverse	GGCTGCACTTGTGATCCTTG
Human *ARRDC2*	forward	GAGGTGGTAGCCGACACTGA
	reverse	CGGTAGCGGAACTCTTGGAT
Human *ARRDC3*	forward	TTTGCCACTTGTCATCGGTA
	reverse	GGTGCTTCAGGTCTTTCAGG
Human *ARRDC4*	forward	TTGATGCTCGAACTGCCATT
	reverse	TGGTGCTTCAGGCTGCTCT
Human *ARRDC5*	forward	CCGTTGCTGCTGTCCGTGAG
	reverse	CTGAGGCGCTGGTGATGATGATG
Human *TXNIP*	forward	CCTGAAGATCACCGATTGGA
	reverse	GGTGGTGGCATGAACTTGAA
Human *CXCL2*	forward	CTCAAGAATGGGCAGAAAGC
	reverse	CTTCAGGAACAGCCACCAAT
Human *SP1*	forward	CCAATGGCTGGCAGATCA
	reverse	CCACCAGAGACTGTGCGATT

For the probes, a FAM fluorescent reporter is coupled to the 5' end, and a TAMRA quencher is coupled to the 3' end.

SupplementalTable 4.siRNAs**used in the present study**

Name		Sequence 5′→3′
si*ARRDC3*	sense	GCUGGACUGAAUCUAGAAATT
	antisense	UUUCUAGAUUCAGUCCAGCTT
si*PAR1*	sense	GGUCUGAAUUGUGUCGCUUTT
	antisense	AAGCGACACAAUUCAGACCTT
si*SP1*#1	sense	CCUCACAGCCACACAACUUTT
	antisense	AAGUUGUGUGGCUGUGAGGTT
si*SP1*#2	sense	CAUACCAGGUGCAAACCAATT
	antisense	UUGGUUUGCACCUGGUAUGTT
